# A combination of plasma phospholipid fatty acids and its association with incidence of type 2 diabetes: The EPIC-InterAct case-cohort study

**DOI:** 10.1371/journal.pmed.1002409

**Published:** 2017-10-11

**Authors:** Fumiaki Imamura, Stephen J. Sharp, Albert Koulman, Matthias B. Schulze, Janine Kröger, Julian L. Griffin, José M. Huerta, Marcela Guevara, Ivonne Sluijs, Antonio Agudo, Eva Ardanaz, Beverley Balkau, Heiner Boeing, Veronique Chajes, Christina C. Dahm, Courtney Dow, Guy Fagherazzi, Edith J. M. Feskens, Paul W. Franks, Diana Gavrila, Marc Gunter, Rudolf Kaaks, Timothy J. Key, Kay-Tee Khaw, Tilman Kühn, Olle Melander, Elena Molina-Portillo, Peter M. Nilsson, Anja Olsen, Kim Overvad, Domenico Palli, Salvatore Panico, Olov Rolandsson, Sabina Sieri, Carlotta Sacerdote, Nadia Slimani, Annemieke M. W. Spijkerman, Anne Tjønneland, Rosario Tumino, Yvonne T. van der Schouw, Claudia Langenberg, Elio Riboli, Nita G. Forouhi, Nick J. Wareham

**Affiliations:** 1 Medical Research Council Epidemiology Unit, University of Cambridge, Cambridge, United Kingdom; 2 National Institute for Health Research Biomedical Research Centres Core Nutritional Biomarker Laboratory, University of Cambridge, Addenbrooke’s Hospital, Cambridge, United Kingdom; 3 National Institute for Health Research Biomedical Research Centres Core Metabolomics and Lipidomics Laboratory, University of Cambridge, Addenbrooke’s Hospital, Cambridge, United Kingdom; 4 Medical Research Council Epidemiology Unit Elsie Widdowson Laboratory, Cambridge, United Kingdom; 5 Department of Molecular Epidemiology, German Institute of Human Nutrition, Potsdam, Germany; 6 Department of Biochemistry, University of Cambridge, Cambridge, United Kingdom; 7 Department of Epidemiology, Consejería de Sanidad y Política Social, CIBER de Epidemiología y Salud Pública, Murcia, Spain; 8 CIBER Epidemiología y Salud Pública, Madrid, Spain; 9 Navarre Public Health Institute, Pamplona, Navarra Institute for Health Research (IdiSNA), Pamplona, Spain; 10 Julius Center for Health Sciences and Primary Care, University Medical Center Utrecht, Utrecht University, Utrecht, Netherlands; 11 Unit of Nutrition, Environment and Cancer, Cancer Epidemiology Research Program, Catalan Institute of Oncology, Barcelona, Spain; 12 Center for Research in Epidemiology and Population Health, Inserm U1018, Paris-Sud University, University Versailles Saint-Quentin-en-Yvelines, Paris Saclay University, Villejuif, France; 13 International Agency for Research on Cancer, Lyon, France; 14 Section for Epidemiology, Department of Public Health, Aarhus University, Aarhus, Denmark; 15 Gustave Roussy Institute, Villejuif, France; 16 Department of Agrotechnology and Food Sciences, Wageningen University, Wageningen, Netherlands; 17 Family Medicine, Department of Public Health and Clinical Medicine, Umeå University, Umeå, Sweden; 18 Department of Clinical Sciences, Lund University, Skane University Hospital, Malmo, Sweden; 19 Department of Epidemiology, Murcia Regional Health Council, Murcia, Spain; 20 Murcia BioHealth Research Institute–Hospital Virgen de la Arrixaca, Murcia, Spain; 21 Division of Cancer Epidemiology, German Cancer Research Center (DKFZ), Heidelberg, Germany; 22 Cancer Epidemiology Unit, Nuffield Department of Population Health, University of Oxford, Oxford, United Kingdom; 23 Department of Public Health and Primary Care, University of Cambridge School of Clinical Medicine, Cambridge, United Kingdom; 24 Escuela Andaluza de Salud Pública, Instituto de Investigación Biosanitaria ibs.GRANADA, Hospitales Universitarios de Granada/Universidad de Granada, Granada, Spain; 25 Danish Cancer Society Research Center, Copenhagen, Denmark; 26 Department of Cardiology, Aalborg University Hospital, Aarhus, Denmark; 27 Cancer Risk Factors and Lifestyle Epidemiology Unit, Cancer Research and Prevention Institute, Florence, Italy; 28 Dipartimento di Medicina Clinica e Chirurgia, Università degli Studi di Federico II University, Naples, Italy; 29 Epidemiology and Prevention Unit, Fondazione IRCCS Istituto Nazionale dei Tumori, Milan, Italy; 30 Unit of Cancer Epidemiology, Città della Salute e della Scienza Hospital–University of Turin and Center for Cancer Prevention, Torino, Italy; 31 National Institute for Public Health and the Environment, Bilthoven, Netherlands; 32 Affiliation Cancer Registry, Department of Prevention, Azienda Sanitaria Provinciale di Ragusa, Ragusa, Italy; 33 School of Public Health, Imperial College London, London, United Kingdom; Chinese University of Hong Kong, CHINA

## Abstract

**Background:**

Combinations of multiple fatty acids may influence cardiometabolic risk more than single fatty acids. The association of a combination of fatty acids with incident type 2 diabetes (T2D) has not been evaluated.

**Methods and findings:**

We measured plasma phospholipid fatty acids by gas chromatography in 27,296 adults, including 12,132 incident cases of T2D, over the follow-up period between baseline (1991–1998) and 31 December 2007 in 8 European countries in EPIC-InterAct, a nested case-cohort study. The first principal component derived by principal component analysis of 27 individual fatty acids (mole percentage) was the main exposure (subsequently called the fatty acid pattern score [FA-pattern score]). The FA-pattern score was partly characterised by high concentrations of linoleic acid, stearic acid, odd-chain fatty acids, and very-long-chain saturated fatty acids and low concentrations of γ-linolenic acid, palmitic acid, and long-chain monounsaturated fatty acids, and it explained 16.1% of the overall variability of the 27 fatty acids. Based on country-specific Prentice-weighted Cox regression and random-effects meta-analysis, the FA-pattern score was associated with lower incident T2D. Comparing the top to the bottom fifth of the score, the hazard ratio of incident T2D was 0.23 (95% CI 0.19–0.29) adjusted for potential confounders and 0.37 (95% CI 0.27–0.50) further adjusted for metabolic risk factors. The association changed little after adjustment for individual fatty acids or fatty acid subclasses. In cross-sectional analyses relating the FA-pattern score to metabolic, genetic, and dietary factors, the FA-pattern score was inversely associated with adiposity, triglycerides, liver enzymes, C-reactive protein, a genetic score representing insulin resistance, and dietary intakes of soft drinks and alcohol and was positively associated with high-density-lipoprotein cholesterol and intakes of polyunsaturated fat, dietary fibre, and coffee (*p <* 0.05 each). Limitations include potential measurement error in the fatty acids and other model covariates and possible residual confounding.

**Conclusions:**

A combination of individual fatty acids, characterised by high concentrations of linoleic acid, odd-chain fatty acids, and very long-chain fatty acids, was associated with lower incidence of T2D. The specific fatty acid pattern may be influenced by metabolic, genetic, and dietary factors.

## Abstract 2

Fumiaki Imamura and colleagues reveal profiles of plasma phospholipid fatty acids that are associated with risk of type 2 diabetes using data from the EPIC cohort.

## Author summary

**Why was this study done?:**

- Fatty acid subclasses (e.g., saturated fatty acids or omega-6 fatty acids) and individual fatty acids in the blood have been studied to understand the aetiology of type 2 diabetes and as biomarkers of dietary intakes.
- Existing studies suggest that different types of fatty acids are mutually correlated and are altered together by pharmacological intervention, dietary intervention, or both.
- However, no study to our knowledge has reported whether a certain combination of different types of circulating individual fatty acids could be associated with the risk of type 2 diabetes.

**What did the researchers do and find?:**

- We evaluated 27 individual fatty acids in the blood samples of adults within a large study from 8 countries in Europe, among a reference sub-cohort sample of 15,919 adults and among 12,132 adults who subsequently developed type 2 diabetes over a follow-up period of 12 years on average.
- We identified a fatty acid combination that was partly represented by a combination of high concentrations of linoleic acid (the most abundant omega-6 polyunsaturated fatty acid), low concentrations palmitic acid (the major saturated fatty acid), and varying concentrations of the other essential and non-essential fatty acids.
- We found that incidence of type 2 diabetes was lower by 63% on average when comparing the 20% of adults with fatty acid profiles most consistent with this particular combination (e.g., high linoleic acid and low palmitic acid) with the 20% of adults with fatty acid profiles least like this particular combination (e.g., low linoleic acid and high palmitic acid).
- The combination of fatty acids was also linked to genes related to insulin resistance, cardiometabolic risk factors, and dietary intakes of polyunsaturated fatty acids, coffee, soft drinks, and dietary fibre.

**What do these findings mean?:**

- A combination of fatty acids may be potentially important in the development of type 2 diabetes over and above individual fatty acids or fatty acid subclasses.
- Dietary, pharmacological, and genetic investigations are warranted to characterise the clinical and biological implications of the combination of different types of individual fatty acids, for example, to predict the risk of type 2 diabetes, to better understand the aetiology of type 2 diabetes, and to consider interventions to favourably alter fatty acid profiles.

## Introduction

Fatty acids play vital roles in metabolic homeostasis, serving as precursors of signalling molecules, energy sources, and constituents of membranes and functional lipids [1,2]. Reflecting their diverse roles, fatty acids have been evaluated as markers of physiological homeostasis, metabolic disorders, and dietary exposure in biological, clinical, and population-based research [3–5]. For example, blood or tissue levels of omega-3 polyunsaturated fatty acids (PUFAs) have been studied as a cardio-protective factor in biochemical and clinical research and as a biomarker of dietary consumption of omega-3 PUFAs in epidemiological research [1,2,4–6]. However, research to date has largely evaluated individual fatty acids or single subgroups of fatty acids, rather than combinations of fatty acids, in terms of mechanism or as potential biomarkers.

Combinations of fatty acids may have aetiological and clinical implications for metabolic diseases including type 2 diabetes (T2D). Insulin resistance and pancreatic lipotoxicity have been found to be influenced by multiple fatty acids. For example, palmitic acid (16:0) induces lipotoxicity, and unsaturated fatty acids may prevent it [7–9]. Pharmacological and nutritional research also warrant considering multiple fatty acids together. Interventions of lipid-lowering drugs or dietary carbohydrates or fats, for example, alter blood concentrations of individual PUFAs and saturated fatty acids (SFAs) jointly [3,9–11]. These findings support the notion that combinations of fatty acids are important to study in relation to the aetiology of T2D and to predict T2D risk.

A few epidemiological studies have identified combinations of circulating or tissue fatty acids associated with adiposity, hypertension, and risks of metabolic syndrome and cardiovascular diseases using a statistical pattern-recognition approach [12–15]. These studies have indicated potential biological and clinical importance of combinations over and above that of individual fatty acids. However, a combination of fatty acids has never been evaluated as a potential risk factor for incident T2D. Thus, we first aimed to identify 1 or more combinations of phospholipid fatty acids that explained variability in multiple fatty acid concentrations, using epidemiological data from the European Prospective Investigation into Cancer and Nutrition (EPIC)–InterAct study. Then, focussing on the single combination of fatty acids that explained the greatest variability, we tested the hypothesis that the combination is associated with the incidence of T2D. To provide mechanistic insights, we further examined the association of this combination of fatty acids with metabolic risk factors, genetic predisposition to obesity and insulin resistance, and dietary intakes in EPIC-InterAct. For metabolic and dietary factors, external validation was performed by evaluating data of the US National Health and Nutrition Examination Survey (NHANES).

## Methods

### Study population

We conducted this work as a substudy of the fatty acid project in EPIC-InterAct to explore a combination of fatty acids to add to our previous work on individual fatty acids and subclasses (S1 Protocol) [16,17]. EPIC-InterAct is a prospective study nested within 8 European countries of the EPIC study (Denmark, France, German, Italy, Netherlands, Spain, Sweden, and UK) [16,18]. In EPIC-InterAct, the case-cohort design was adopted to combine the advantages of a prospective design with the efficiency of a case-control design [19]. From the 340,234 adults with 3.99 million person-years of follow-up of the EPIC study, EPIC-InterAct (1) randomly selected 16,835 adults (‘sub-cohort’) and (2) identified 12,403 incident cases of T2D occurring by 31 December 2007; the identified cases included 778 cases in the sub-cohort by design (S1 Fig) [16,18]. All participants gave written informed consent. The study was approved by local ethics committees and the institutional review board of the International Agency for Research on Cancer [18].

The current study included 15,919 adults from the sub-cohort—after excluding 916 meeting 1 or more exclusion criteria: prevalent diabetes (*n* = 548), missing information on fatty acids (*n* = 156), missing information on incident T2D (*n* = 129), and post-censoring T2D (*n* = 4)—and included 12,132 incident T2D cases, after excluding 271 adults missing information on fatty acids (S1 Fig). In summary, we evaluated 27,296 adults in this study (12,132 cases, including 755 cases from the sub-cohort; and 15,919 adults from the sub-cohort).

### Ascertainment of type 2 diabetes

Prevalent diabetes cases (excluded from the study) were identified by baseline self-report of a diagnosis, physician’s diagnosis, anti-diabetic drug use, or other evidence of T2D before the baseline date in EPIC-InterAct [18]. Incident T2D was ascertained from multiple information sources reviewed by each participating centre [18]: self-report, linkage to primary-care registers, secondary-care registers, medication use (drug registers), hospital admissions, and mortality data. Information from any follow-up visit or external evidence with a date later than the baseline visit was used. In Denmark and Sweden, incident cases were identified via local and national diabetes and pharmaceutical registers, and hence all ascertained cases were considered to be verified. Follow-up was to the date of diagnosis, 31 December 2007, or the date of death, whichever occurred earliest.

### Assessment of fatty acids and other variables

We evaluated relative concentrations of 27 individual fatty acids expressed as mole percentage of total plasma phospholipid fatty acids (Table 1), as previously described (S1 Text) [16,20]. These measurements were masked to case status. Thirty-seven fatty acids of plasma phospholipids were quantified by gas chromatography [20]. In the current analysis, 10 fatty acids were excluded because their relative concentrations were <0.05% on average. Coefficients of variation of the 27 fatty acids ranged from 1.9% to 4.6% [20].

**Table 1:** Relative concentrations of plasma phospholipid fatty acids and their correlations with the identified fatty acid pattern score: EPIC-InterAct sub-cohort (*n* = 15,919).

Individual FA*	Name	Percent of total phospholipid FAs		Correlation with the FA-pattern score^†^
		Median	10th and 90th percentiles	
**Long-chain saturated FA**				
14:0	Myristic acid	0.38	0.26, 0.53	−0.34
16:0	Palmitic acid	30.1	28.2, 32.4	−0.51
18:0	Stearic acid	14.1	12.4, 15.8	0.36
**Odd-chain saturated FA**				
15:0	Pentadecanoic acid	0.22	0.15, 0.31	0.27
17:0	Heptadecanoic acid	0.42	0.31, 0.53	0.57
**Very-long-chain saturated FA**				
20:0	Arachidic acid	0.13	0.10, 0.18	0.55
22:0	Behenic acid	0.23	0.17, 0.32	0.69
23:0	Tricosanoic acid	0.11	0.07, 0.16	0.49
24:0	Lignoceric acid	0.22	0.17, 0.30	0.59
**Monounsaturated FA**				
16:1	Palmitoleic acid	0.47	0.29, 0.79	−0.75
18:1n-9	Oleic acid	9.6	7.7, 11.9	−0.50
**Very-long-chain monounsaturated FA**				
20:1	Gondoic acid	0.25	0.17, 0.34	−0.04
24:1	Nervonic acid	0.34	0.25, 0.46	0.42
**Omega-6 PUFA**				
18:2n-6	Linoleic acid	22.5	18.4, 26.6	0.45
18:3n-6	γ-linolenic acid	0.07	0.02, 0.14	−0.51
20:3n-6	Dihomo-γ-linolenic acid	3.1	2.2, 4.2	−0.38
20:4n-6	Arachidonic acid	9.2	7.0, 11.7	−0.17
**Omega-3 PUFA**				
18:3n-3	α-linolenic acid	0.28	0.15, 0.54	−0.04
20:5n-3	Eicosapentaenoic acid	1.02	0.52, 2.13	0.03
22:5n-3	Docosapentaenoic acid	0.92	0.62, 1.22	0.02
22:6n-3	Docosahexaenoic acid	4.1	2.7, 5.9	0.23
**Trans unsaturated FA**				
Trans 18:1	Elaidic acid	0.21	0.10, 0.52	0.20
Trans 18:2	Trans linoleic acid	0.07	0.04, 0.09	0.18
**Other**				
17:1	Heptadecenoic acid	0.06	0.00, 0.13	−0.13
20:2	Eicosadienoic acid	0.38	0.30, 0.47	−0.08
22:4	Adrenic acid	0.28	0.20, 0.39	−0.39
22:5n-6	Osbond acid	0.19	0.11, 0.31	−0.39

*FAs are subclassified according to generic classification.

^†^The first principal component (FA-pattern score) derived by principal component analysis of the 27 individual fatty acids (*n* = 15,919), used as the main exposure variable in this study. Coefficients to calculate the FA-pattern score are presented in S5 Table.

FA, fatty acid; FA-pattern score, fatty acid pattern score; PUFA, polyunsaturated fatty acid.

At baseline, weight, height, and waist circumference were measured directly in every centre. Waist circumference was not measured in Umea, Sweden (*n* = 1,845) [18]. Sociodemographic factors, smoking status, and medical history were assessed by a questionnaire for general health. Physical activity was assessed by a questionnaire validated previously [21]. Dietary variables were derived centrally based on food frequency questionnaires or diet histories standardised in each cohort [22,23]. Using blood samples stored at −196°C (or −150°C in Denmark), biochemical assays were performed at Stichting Ingenhousz Laboratory, Etten-Leur, Netherlands, for glucose, triglycerides, high-density lipoprotein cholesterol (HDL-C), triglycerides, high-sensitivity C-reactive protein (hsCRP), and proteins related to hepatic function—alanine transaminase (ALT), γ-glutamyl transferase (GGT), and aspartate transaminase (AST)—as the liver is the major organ metabolising fatty acids.

Genetic information became available in 22,179 adults with fatty acid data, assayed with Illumina Human660W-Quad BeadChip (Illumina, Little Chesterford, UK; *n* = 9,166) and MetaboChip (Illumina; *n* = 13,013) [24]. Using these data, we conducted post hoc analyses to examine whether genetic predisposition to metabolic risk was associated with the FA-pattern score. We calculated weighted genetic risk scores for body mass index (BMI) (*n* loci = 97) [25] and for insulin resistance (*n* loci = 10) [24] using published measures of genome-wide associations (S1 Text).

### Derivation of fatty acid pattern score

Principal component analysis (PCA) was performed in the sub-cohort (*n* = 15,919) to combine multiple fatty acids (Table 1) together in a way to explain as much variation of those fatty acids as possible. Sampling weights were applied so that each of the 8 countries equally contributed to the PCA. Eigenvalues divided by 27 were assessed as percent of variance explained.

**Fig 1:**
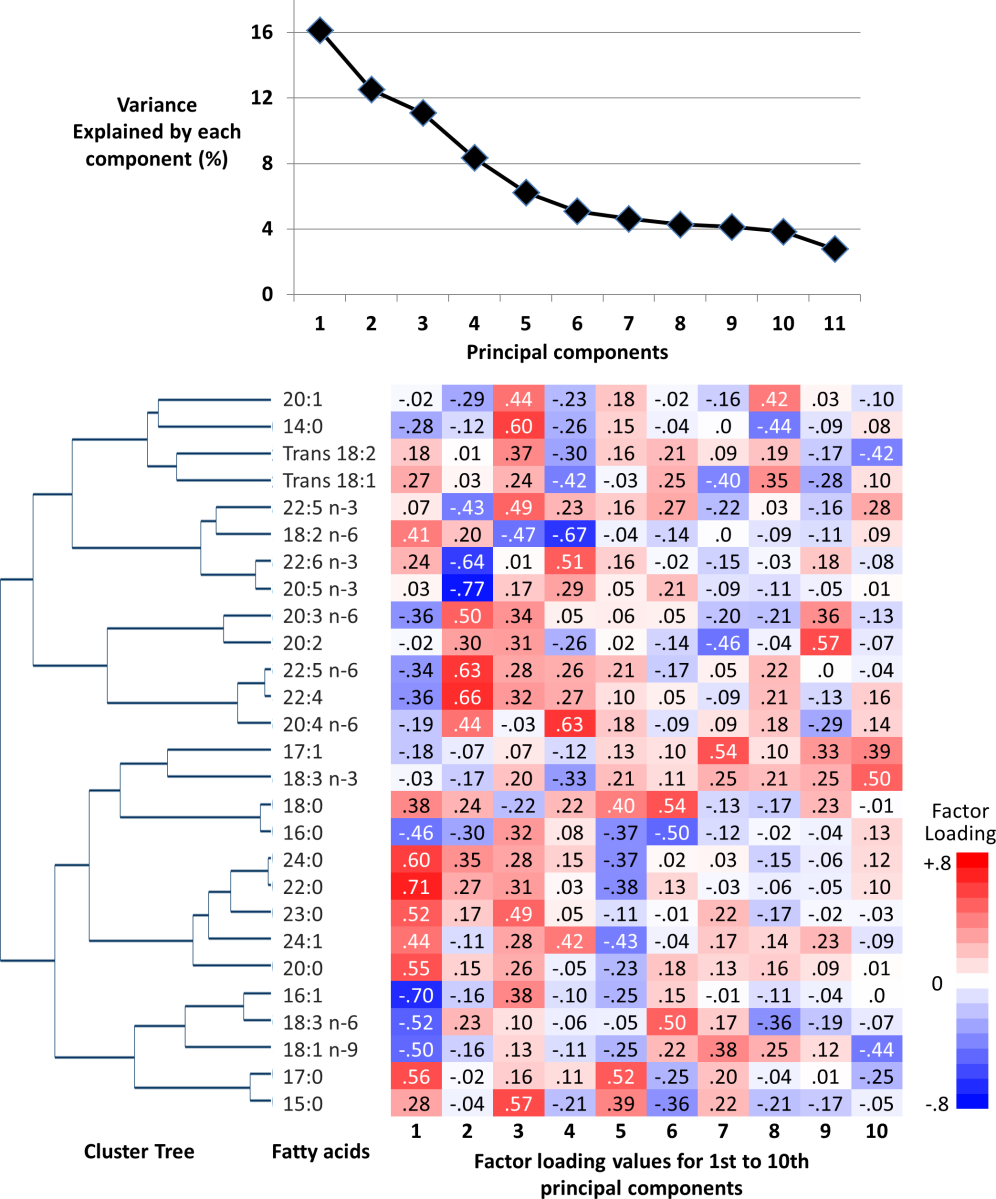
Principal components and clusters of 27 fatty acids in the EPIC-InterAct sub-cohort (*n* = 15,919). Top: The proportion of total variance of 27 fatty acids explained by each principal component. Bottom: Hierarchical cluster tree on the left and factor loadings (measures of contributions of fatty acids to principal components) on the right.

Principal components were inferred as representing fatty acid patterns. The pattern matrix from PCA was then used to calculate the scores, referred to as FA-pattern scores, among the rest of the study population (incident T2D cases not in the sub-cohort, Fig 1) and was also applied to quality control samples (*n* = 860) to assess the contribution of any batch effects [20]. We chose to focus on the first principal component for further aetiological analyses to provide potential biological implications of this single combination of fatty acids. This enabled us to conduct and report a detailed investigation into the associations of this combination with T2D incidence and metabolic, dietary, and genetic variables, and their biological implications; it also removed the need for subjective decisions about how many components to derive, which matrix rotation method to use, and how to account for multiple testing [26].

### Statistical analysis

All analyses were performed using Stata (StataCorp, College Station, Texas, US), with α_two-sided_ = 0.05. For descriptive purposes, a hierarchical cluster tree was generated to visually assess correlation between fatty acids [27]. Pearson correlation coefficients between fatty acids were also calculated. The strength of association of the FA-pattern score with incident T2D was evaluated by estimating hazard ratios (HRs) and 95% CIs from Prentice-weighted Cox regression, with age as the underlying timescale [19]. The estimates were obtained in each country and pooled by random-effects meta-analysis [28] for quintiles specific to the sub-cohort, for a continuous term per interdecile range (the difference between the 90th and 10th percentiles of the distribution), and for cubic-spline terms to test non-linear associations [29]. We additionally computed a 95% predicted interval for the primary results by combining random-effects variation (tau^2^) and variation of the main estimate [28].

The models included potential confounders, including demographics, prevalent heart disease and stroke, medication use, smoking status, physical activity, and dietary factors (consumption of alcohol, soft drinks, dietary fibre, fruits, vegetables, and processed meats), that are associated with cardiometabolic health in general. We also adjusted for BMI, waist circumference, glucose, lipids, hsCRP, and liver enzymes to examine their influence on the associations of interest. Potential confounding by genetic predisposition for greater BMI and insulin resistance was also assessed.

In pre-specified analyses, we examined whether observed associations varied by baseline age, sex, and BMI, testing an interaction term for each factor and the FA-pattern score in regression analysis. Effect modification by blood triglycerides, use of lipid-lowering drugs (yes or no), and alcohol consumption (consumer or non-consumer) was also tested post hoc because of the association of triglycerides with the FA-pattern score (*r* = −0.29) and possible effects of lipid-lowering drugs and alcohol on de novo lipogenesis. Missing covariates were imputed by country, using multiple imputation by chained equations with variables for the FA-pattern score, covariates, survival time, and case status [30]. We report results from single imputation, because between-imputation variability was <0.2% of total variability in multiple imputation (20 datasets); we performed sensitivity analysis using multiple imputation and complete-case analysis.

As a sensitivity analysis to assess whether HR varied over the follow-up time by reverse causation, stratified analysis was performed by splitting follow-up time at 7 years after baseline and by censoring any events occurring within the first 2 years as non-cases. We additionally evaluated the stability of our findings: examining the consistency of a main finding for the single principal component when PCA was performed after Box–Cox transformation, improving normality of distribution of all fatty acid variables. We also examined whether or not the main result was driven by single fatty acids or fatty acid subclasses through 2 approaches: adjusting models for single fatty acids and subclasses separately, and repeating the analysis after PCA of fatty acids excluding each of the 27 fatty acids or subclasses one at a time. We performed internal cross-validation [31]: First, we re-derived the FA-pattern score in a subset selected by country, age, sex, and BMI (test set); second, we applied the scoring matrix to another subset (validation set) to derive the FA-pattern score, and then we examined the associations of the independently derived score with incident T2D.

### Analysis of metabolic, genetic, and dietary factors

To investigate potential mechanisms for the association of the FA-pattern score with incident T2D, we estimated cross-sectional associations of the FA-pattern score with each of selected metabolic risk factors (BMI, waist circumference, lipids, glucose, hsCRP, and liver enzymes) using linear regression. Additionally, modified Poisson regression [32] was used to examine the cross-sectional association of the FA-pattern score with prevalence of hepatic steatosis defined as ALT greater than cut-points previously validated against ultrasound (30 U/l for men, 19 U/l for women) [33]. We further fitted linear regression to assess whether genetic risk scores for BMI and for insulin resistance (independent variables) could explain variability in the FA-pattern score (dependent variable). These regression models statistically adjusted for age and the other covariates used for longitudinal analyses.

We also evaluated dietary factors as potential lifestyle determinants of the FA-pattern score. Multivariable-adjusted linear models included dietary determinants as independent variables and the FA-pattern score (scaled to 1 standard deviation) as a dependent variable. This analysis evaluated major macronutrient and fibre intakes (nutrient-based analysis) and 18 selected foods or beverages (food-based analysis).

### Assessment of external validity

Recognising the risk of false-positive findings based on our data-driven approach, we conducted post hoc assessment of the external validity of the FA-pattern score derived in EPIC-InterAct, using cross-sectional data from NHANES 2003–2004 (*n* = 1,566) on total plasma fatty acids, metabolic factors, dietary factors, and potential confounders. Using the scoring matrix derived from EPIC-InterAct, we calculated the FA-pattern score in NHANES (S2 Text) [15]. Using linear regression adjusting for potential confounders, replication analyses were performed (S3 Text). In dietary analyses, 18 dietary items were first assessed in EPIC-InterAct with backward variable selection (*p* = 0.2 as a cutoff, additionally for the purpose of adjustment [34]) to identify which dietary variables predicted the FA-pattern score together. Then we tested selected dietary factors in NHANES for external validation (S3 Text) in linear regression adjusting for potential confounders and including the same dietary variables. The 18 food groups first tested in EPIC-InterAct were selected by possible biology of diets, fatty acid profiles, and T2D, and evaluated both individually and simultaneously.

## Results

The first component derived by PCA explained 16.1% of the variation of 27 fatty acids, and 6 to 10 components explained more variation than 1 fatty acid could explain (>3.7% of total; ‘eigenvalue’ > 1.0) (Fig 1). The first 4 components had loading values (e.g., >0.6 or <−0.6) in multiple fatty acid classes. Selected to gain insight into the biological importance of a combination of fatty acids, the first component reflected relationships between fatty acids varying in chain length and degree of unsaturation, including fatty acids that can be synthesised endogenously and those derived from dietary consumption (Table 1; Fig 1). A similar pattern was identified in cluster analysis, as fatty acids adjacent in the tree had similar loading values (Fig 1). Major contributors (correlation coefficients *r >* 0.5 or *r <* −0.5) were palmitic acid (16:0, *r* = −0.51), palmitoleic acid (16:1, *r* = −0.75), and γ-linolenic acid (18:3n-6, *r* = −0.51). Heptadecanoic acid (17:0) and very-long-chain SFAs (VLSFAs) with 20 or more carbons had positive contributions (*r* = 0.5–0.7), but their relative concentrations were low (<1% of total). While linoleic acid (18:2n-6) had a positive contribution (*r* = 0.45), the other PUFAs, and trans unsaturated fatty acids had lower contributions (−0.25 < *r <* 0.25) (Fig 1). The coefficient of variation of the FA-pattern score was 6.0% based on the quality control samples.

Adults with higher FA-pattern score were more likely to be women, non-smokers, non-users of lipid-lowering drugs, and those with generally healthier profiles of metabolic risk factors, while there was no significant relationship with age or education (S1 Table). Covariates had missing values in <5% of adults, except 49.9% for family history of diabetes, which was not assessed in 12 of the 26 study centres (S2 Table). Where it was assessed, 24.5% of participants had missing information.

### Association of the fatty acid pattern score with incidence of type 2 diabetes

**Fig 2:**
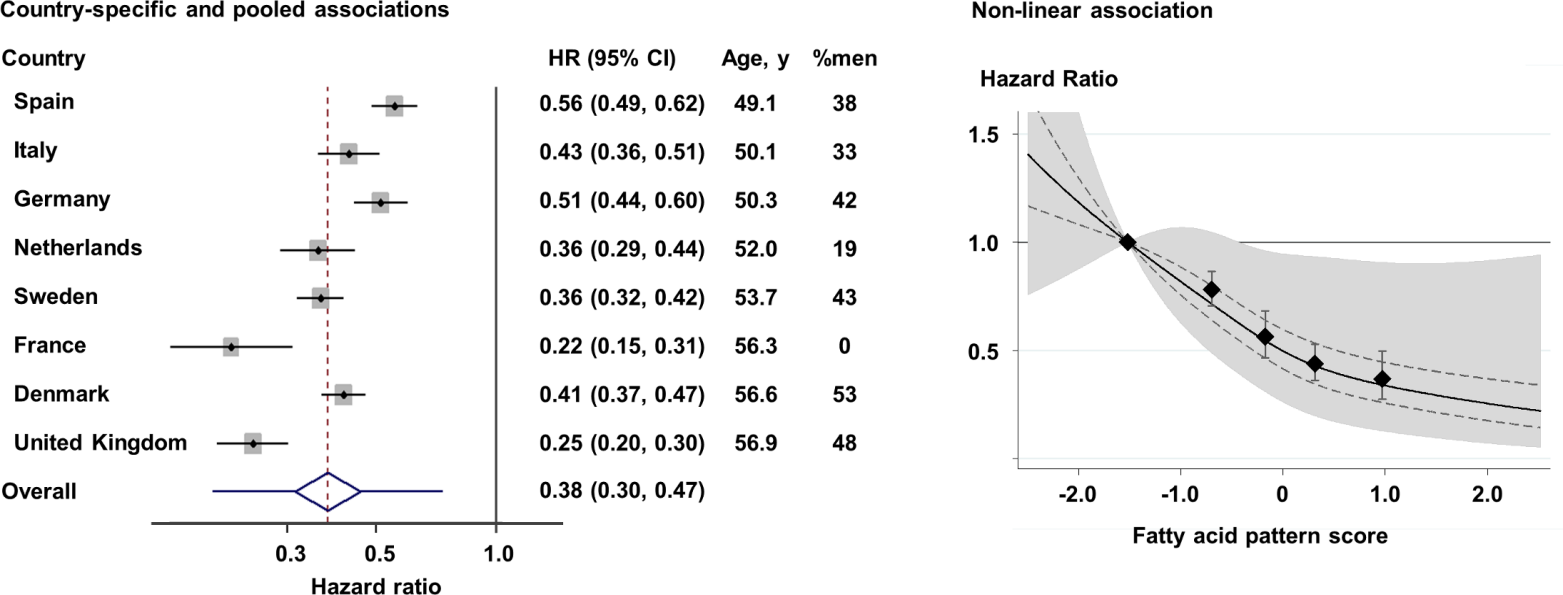
Prospective associations of the fatty acid pattern score with incident diabetes in 8 countries: EPIC-InterAct (*n* = 27,296). Left: Hazard ratios (HRs) per country-specific range of 10th to 90th percentiles (*p* for heterogeneity by age and sex = 0.005 and 0.02, respectively), and pooled by random-effects meta-analysis. The diamond and error bars of the pooled estimate represent the 95% confidence interval and predicted interval (0.20 to 0.74), respectively. Right: HR based on quintiles and restricted cubic spline (*p* non-linearity = 0.001) (solid line). Error bars in both panels, and dotted lines in the right panel, indicate the 95% confidence intervals of HRs, and the shaded area in the right panel is the predicted interval. All analyses adjusted for covariates as in the most adjusted model in Table 2.

In the longitudinal analysis of 12,132 cases per 190,148.9 person-years (11.9 y of follow-up per person on average), the FA-pattern score was strongly associated with incident T2D. Adjusted for sociodemographic variables, dietary factors, and medical history, the HR (95% CI) of T2D comparing the top to the bottom fifth of the FA-pattern score was 0.23 (0.19–0.29) (*p* trend < 0.001) (Table 2). The association persisted after adjustment for BMI (HR 0.32; 95% CI 0.25–0.40) and for triglycerides and HDL-C (0.37; 95% CI 0.27–0.50). Results changed little when additionally adjusted for concentrations of random glucose, hsCRP, hepatic enzymes, other dietary factors, family history of T2D, and genetic risk scores for obesity and insulin resistance (S3 Table). The association varied across the 8 countries (Fig 2; *I*^2^ = 88%); this variation was partly explained by country-specific mean ages and percentage of men (*p <* 0.05 each), although an inverse association was observed in all countries.

**Table 2:** Association of the fatty acid pattern score with incidence of type 2 diabetes: EPIC -InterAct (*n* = 27,296).

Model	Quintile of the fatty acid pattern score*					*P* trend
	I	II	III	IV	V	
**Number of cases**	4,277	2,910	2,113	1,587	1,245	
**Incidence rate per 100,000 person-years**^**†**^	679	476	349	283	219	<0.001
**Pooled hazard ratio (95% CI)**^**‡**^						
Multivariable-adjusted	1.0 (reference)	0.68 (0.62–0.75)	0.46 (0.40–0.53)	0.32 (0.28–0.35)	0.23 (0.19–0.29)	<0.001
+ Body mass index	1.0 (reference)	0.75 (0.69–0.82)	0.53 (0.46–0.62)	0.40 (0.35–0.46)	0.32 (0.25–0.40)	<0.001
+ Triglycerides and HDL-C	1.0 (reference)	0.78 (0.70–0.86)	0.56 (0.46–0.68)	0.44 (0.36–0.53)	0.37 (0.27–0.50)	<0.001

*Five categories were obtained by quintiles of the fatty acid pattern score in EPIC-InterAct. Each participant was assigned a fatty acid pattern score (mean = 0, standard deviation = 1) by principal component analysis using 27 individual fatty acids.

^†^Incidence was calculated in the random sub-cohort (*n* = 15,919).

^‡^Multivariable-adjusted Prentice-weighted Cox regression models. The first model adjusted for recruitment centre (2 to 6 categories in each country), age as covariate, and underlying timescale, sex, education history, smoking status, alcohol consumption, dietary factors (dietary fibre, fruits, vegetables, processed meats, soft drinks), physical activity, menopause status, hormone replacement use, and prevalent diseases (myocardial infarction or angina, stroke, hypertension, and dyslipidaemia). Pooled results from 8 countries were obtained by random-effects meta-analysis. Hazard ratios (95% CIs) per interdecile range in the 3 models were 0.28 (0.24–0.33), 0.33 (0.28–0.40), and 0.38 (0.30–0.47), respectively.

HDL-C, high-density lipoprotein cholesterol.

There was no evidence of effect modification by baseline age, sex, BMI, triglycerides, lipid-lowering drug use, or alcohol consumption (*p* interaction > 0.1 each). The main result was stable in sensitivity analyses that explored the influence of imputation, duration of follow-up, and normality of distribution (S3 Table). In analyses adjusting for individual fatty acid variables (S2 Fig), adjustment for 17:0 attenuated the estimates to the greatest extent among the fatty acid variables we evaluated, shifting the HR (95% CI) of 0.38 (0.30–0.47) to 0.53 (0.42–0.67), but with the association remaining significant. Cross-validation analysis confirmed the stability of the findings (S4 Table). For instance, when the FA-pattern score was derived in 7 countries, not 8, and the scoring algorithm was applied to adults in the 1 country excluded, the summary HR (95% CI) in the most adjusted model was 0.40 (0.34–0.50).

### Association of the fatty acid pattern score with metabolic, genetic, and dietary factors

In both EPIC-InterAct and NHANES (see S5 Table for scoring coefficients), the FA-pattern score was associated with metabolic risk factors in the direction consistent with the above findings for incident T2D. Inverse associations were seen with BMI, triglycerides, glucose, hsCRP, ALT, AST, GGT, and the likelihood of having hepatic steatosis (*p <* 0.001 each) (Table 3). A significant positive association with HDL-C was observed in NHANES (*p <* 0.001), but not in EPIC-InterAct (*p* = 0.7).

**Table 3:** Associations of the fatty acid pattern score with metabolic factors in EPIC-InterAct and with metabolic factors in the US NHANES 2003–2004.

Metabolic factor	EPIC-InterAct (*n* = 15,919)	NHANES (*n* = 1,566)
Body mass index, kg/m^2^	−1.2 (−1.5, −0.9)	−2.3 (−3.0, −1.7)
Triglycerides, mmol/l	−0.6 (−0.7, −0.5)	−1.9 (−2.2, −1.6)
HDL-C, mmol/l	0.00 (−0.03, 0.02)	0.26 (0.21, 0.31)
Glucose, mmol/l	−0.24 (−0.29, −0.19)	−0.26 (−0.37, −0.15)
Alanine transaminase, U/l	−3.2 (−4.2, −2.2)	−3.7 (−6.1, −1.3)
Aspartate transaminase, U/l	−3.1 (−4.1, −2.1)	−5.6 (−8.1, −3.2)
γ-glutamyl transferase, U/l	−15.3 (−20.8, −9.9)	−13.5 (−18.5, −8.4)
C-reactive protein, nmol/l	−0.43 (−0.61, −0.26)	−0.15 (−0.26, −0.05)
High risk of hepatic steatosis, percent prevalence*	−28% (−32%, −23%)	−16% (−30%, −1%)

For metabolic risk factors, values are difference (95% confidence interval) in each metabolic factor per interdecile range of fatty acid pattern score. Linear regression analysis was performed, adjusting for potential confounders and body mass index (for metabolic factors except body mass index) (see S3 Text for details). All differences were significant, *p <* 0.02, with exception of HDL-C in EPIC-InterAct (*p* = 0.7).

*Prevalence ratio was estimated for the likelihood of having hepatic steatosis (alanine transaminase > 30 U/l for men and >19 U/l for women [33]) (i.e., relative difference in prevalence).

HDL-C, high-density lipoprotein cholesterol; NHANES, National Health and Nutrition Examination Survey.

In genetic analyses (EPIC-InterAct only), a gene score related to higher BMI was not significantly associated with the FA-pattern score: +0.1% of SD of the FA-pattern score (95% CI −0.6% to +1.7%; *p* = 0.3) per interdecile range of the genetic score. A gene score related to higher insulin resistance was significantly associated with lower FA-pattern score: −1.9% of SD (95% CI −3.4% to −0.4%; *p* = 0.02).

**Fig 3:**
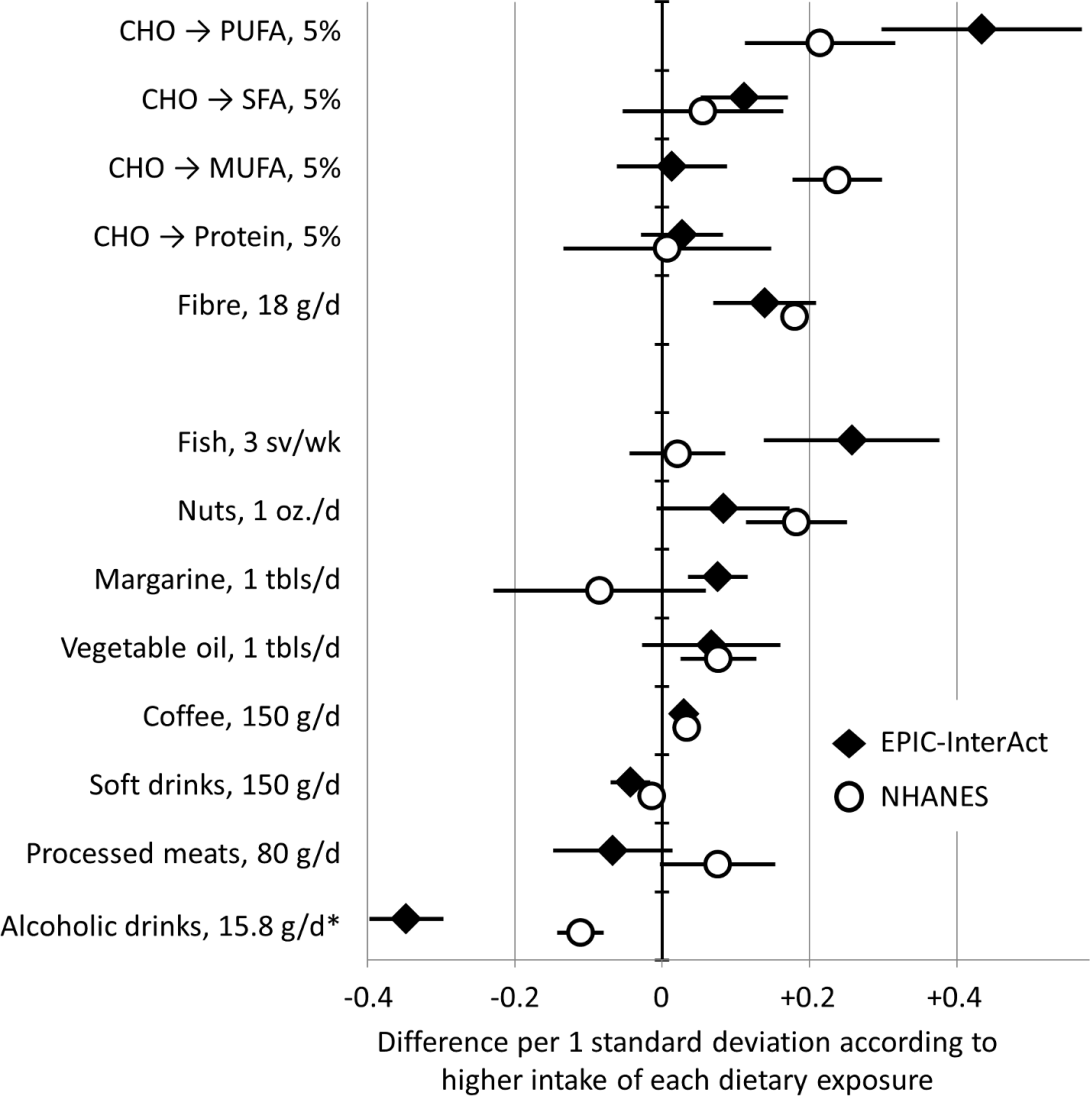
Associations of dietary factors with the fatty acid pattern score in EPIC-InterAct (1991–1998, *n* = 15,566) and the US National Health and Nutrition Examination Survey (2003–2004, *n* = 1,500). Error bars are 95% confidence intervals. In analysis for macronutrients, analysis estimated potential effects of replacing carbohydrate (CHO) intakes with intakes of polyunsaturated fatty acid (PUFA), monounsaturated fatty acid (MUFA), saturated fatty acid (SFA), and protein by the amount of 5% of total caloric intake. Dietary factors were scaled for interpretability: sv, serving; oz., ounce (28.8 g); tbls, tablespoon. *Alcoholic drinks were examined as grams of ethanol.

In dietary analyses in EPIC-InterAct and NHANES, higher intakes of PUFAs and fibre were associated with higher FA-pattern score in both cohorts (Fig 3). For example, replacing carbohydrates with PUFAs in the diet by an amount equivalent to 5% of total energy was positively associated with the FA-pattern score (0.43 SD of the score, 95% CI 0.30–0.57) in EPIC-InterAct and 0.21 (95% CI 0.11–0.32) in NHANES. In food-based analysis of EPIC-InterAct, the FA-pattern score was significantly related to higher intakes of fish, margarine, and coffee and lower intakes of soft drinks and alcoholic beverages (*p <* 0.05 each) when assessed individually (S3 Fig) and simultaneously (Fig 3). In NHANES, findings from EPIC-InterAct for soft drinks, coffee, and alcohol were replicated (*p <* 0.04) (Fig 3).

## Discussion

We evaluated fatty acid profiles among adults in 8 European countries and derived a FA-pattern score that represents a combination of both essential and non-essential fatty acids and that is characterised by high relative concentrations of linoleic acid (18:2n-6), stearic acid (18:0), odd-chain SFAs, and VLSFAs (≥20 carbons), and by low relative concentrations of γ-linolenic acid (18:3n-6), monounsaturated fatty acids (MUFAs), and long-chain SFAs (14:0 and 16:0). The unique combination was associated with dietary, metabolic, and genetic factors, and prospectively associated with a lower incidence of T2D. Comparing the top fifth to the bottom fifth of the FA-pattern score, T2D incidence was lower by approximately 60%. This robust association with incident T2D was independent of established risk factors and also any single fatty acids or fatty acid subclasses. These findings support the hypothesis that a combination of multiple fatty acids is an important marker for the development of T2D above and beyond the roles of single types of fatty acids. The combination of essential and non-essential fatty acids is of strong interest for further clinical or population-based investigations to predict T2D risk, identify interventional agents for T2D prevention, and better understand the aetiology of T2D.

The combination of fatty acids contributing to the identified FA-pattern score fits with known mechanisms involving the de novo lipogenesis (DNL) pathway. In DNL, fatty acids including 14:0, 16:0, 16:1n-7, and 18:1n-9 are synthesised endogenously, where stearoyl-CoA desaturase (SCD) converts 16:0 to 16:1n-7 as a rate-limiting step of fatty acid synthesis. The inverse correlation of 18:2n-6 with these fatty acids may reflect its role as a ligand of peroxisome proliferator-activated receptor α (PPARα) [2,5]. PPARα down-regulates SCD and ELOVL (elongation of very-long-chain fatty acid) enzymes, explaining the observed inverse correlation of 18:2n-6 with MUFAs, 16:0, 18:3n-6, and other n-6 PUFAs [2]. An exception of PPARα’s action is activation of ELOVL3, which leads to synthesis of VLSFAs in the adipose tissue [35] and supports the observed associations between 18:2n-6, 18:0, and VLSFAs. While our findings are in line with the benefit of dietary PUFAs (predominantly 18:2n-6), other major PUFAs (e.g., omega-3 PUFA) contributed little to the primary fatty acid combination. This could reflect their diverse roles in eicosanoid pathways and pro- and anti-inflammatory pathways, and their associations with dietary intakes (e.g., fish) independent of DNL-driving dietary factors [5].

These suggested mechanisms are linked to the development of T2D. Activation of PPARα suppresses hepatic DNL and pro-inflammatory pathways that lead to insulin resistance, dyslipidaemia, and fatty liver [2,5,36]. In an experimental setting, for example, *PPARα* knock-out mice developed fatty liver exhibiting overt hepatic lipogenesis [37]. Main products of DNL include 16:0 and diacylglycerols that cause a pro-inflammatory response, endoplasmic reticulum stress, and insulin resistance [1,38]. Thus, our analysis yielded a combination of multiple fatty acids that may represent biological pathways related to insulin resistance, inflammatory responses, and T2D risk. This was confirmed with the observed associations of FA-pattern score with metabolic risk factors in an expected direction and the association of FA-pattern score with genetic predisposition to insulin resistance. Associations of gene variants with fatty acids and with incident T2D cannot be confounded by long-term lifestyle characteristics. Therefore, the specific gene variants, the identified combination of fatty acids, and the risk of T2D are likely to be on the same causal pathway, warranting future research to elucidate how insulin resistance specifically alters fatty acid profiles or vice versa.

Our analysis and prior studies derived a similar combination of fatty acids [12–15], but no previous studies to our knowledge evaluated T2D incidence as an outcome. Past studies used different methods and examined varied numbers of fatty acids (10 to 42) of phospholipids [15], cholesteryl esters [12,15], plasma [13], or adipose tissue [14]. Despite the differences, all of the studies reported a combination of fatty acids partly driven by higher levels of 18:2n6 with lower 16:0, which could reflect activity of DNL [39]. These combinations were found to be associated with lower blood pressure, greater endothelial function, less weight gain over time, or lower risk of metabolic syndrome [12–14]. In contrast, an association with ischaemic heart disease or stroke was not significant [15]. The inconsistency depending on outcome is predictable as DNL could promote insulin resistance, but suppress atherosclerosis [36]. Statins and other lipid-lowering drugs also alter fatty acid profiles [10] and have divergent effects on heart disease and T2D [40]. Thus, a fatty acid pattern can be a future focus of investigations of cardiometabolic diseases and related interventions.

The association of the FA-pattern score with incident T2D was not fully explained by any single fatty acid, but was partly attenuated by adjustment for odd-chain SFAs and VLSFAs. These SFAs are associated with lower risk of cardiometabolic diseases [16,41,42], while their biological roles remains understudied. High phospholipid VLSFAs may reflect high activity of PPARα, which leads to VLSFA synthesis and less insulin resistance, apoptotic cell death, and pancreatic dysfunction [41,43]. Blood odd-chain SFAs may partly reflect dairy consumption [3,44], gut microbiota [45], or endogenous synthesis through α-oxidation [46], and thus any correlates to those factors could explain our findings. Evidence for these mechanisms and relationships with other fatty acids remains scarce and deserves further investigation.

Dietary correlates with the combination of fatty acids deserve discussion, as they were replicated in EPIC-InterAct and NHANES: alcohol and soft drinks as negative correlates, and coffee, fibre, and PUFAs as positive correlates. The finding for alcohol consumption is likely to reflect its lipogenic effect, a risk factor for liver cirrhosis and T2D [47]. Regarding coffee consumption, polyphenols may deactivate DNL [48] and lower triglyceride levels and T2D risk [49–51]. Increased PUFA intake (predominantly n-6 PUFAs) could increase insulin sensitivity as well as lower DNL [2,5,52]. Our findings for soft drinks and fibre may also reflect their glycaemic and anti-glycaemic effects, respectively, as a high glycaemic effect leads to insulin secretion and DNL [2,53].

Strengths of this work include the standardised assay of fatty acid profiles in an EPIC-InterAct population with geographic diversity; the large study size (to our knowledge by far the largest among studies of fatty acid biomarkers), allowing various sensitivity analyses; and the generalisability of our findings, strengthened by our findings across 8 European countries and the US NHANES. By focussing on a single combination of fatty acids, we were able to report details of its association with incident T2D and metabolic, genetic, and dietary factors. However, this also limited our investigation of other fatty acid patterns, in particular in relation to omega-3 fatty acids. Other limitations include possible residual confounding by factors unmeasured or measured imprecisely, although we adjusted for many covariates including major risk factors for T2D. Whether or not the combination of fatty acids itself caused the T2D onset remains unestablished. Possible exposure misclassification due to single fatty acid measurements and possible outcome misclassification were limitations, but likely to be independent of T2D case status and fatty acid profiles, respectively. We found no strong reason to think that these limitations would alter the overall conclusions. Lastly, the generalisability of our findings might be limited to high-income Western populations, and fatty acid patterns in other populations with diverse genetic backgrounds and dietary patterns are of future interest.

In conclusion, we identified a combination of plasma phospholipid fatty acids characterised by high relative concentrations of 18:2n-6, VLSFAs, and odd-chain SFAs and low relative concentrations of long-chain SFAs and MUFAs, some of which are synthesised endogenously. This particular profile was associated with a 3-fold lower relative risk of incident T2D in European populations after adjustment for confounding. While both genes and diet were linked to the FA-pattern score, association of the FA-pattern score with T2D was independent of established risk factors for T2D and not driven by individual fatty acids. These findings highlight that multiple fatty acids are jointly related to the development of T2D. The combination of fatty acids warrants further investigation of its determinants and potential application as a marker of metabolic characteristics.

## Supporting information

S1 STROBE Checklist (PDF)

S1 Fig Case-cohort study design of EPIC-InterAct and the selection of participants for the current analysis. (PDF)

S2 Fig Association of the fatty acid pattern score with incident type 2 diabetes: sensitivity analysis to examine the influence of single fatty acids and single fatty acid subclasses. (PDF)

S3 Fig Cross-sectional association between dietary consumption of major food groups and the fatty acid pattern score: EPIC-InterAct (*n* = 15,566). (PDF)

S1 Protocol Study protocol. (PDF)

S1 Table Baseline characteristics according to quintile of the fatty acid pattern score in the sub-cohort of EPIC-InterAct (*n* = 15,919). (PDF)

S2 Table Baseline characteristics according to presence or absence of missing information among participants with fatty acid measures: EPIC-InterAct (*n* = 27,296). (PDF)

S3 Table Prospective associations of the fatty acid pattern score with incident type 2 diabetes in EPIC-InterAct: assessment of the influence of subsets of covariates, missing information, duration of follow-up, and normality of fatty acid variables. (PDF)

S4 Table Internal cross-validation for derivation of the fatty acid pattern score and its prospective association with incident type 2 diabetes in EPIC-InterAct: sensitivity analysis to examine internal validity. (PDF)

S5 Table Coefficients to calculate the fatty acid pattern score derived from the sub-cohort of EPIC-InterAct (*n* = 15,919). (PDF)

S1 Text Fatty acid assay and genetic information in EPIC-InterAct. (PDF)

S2 Text External validation of associations of the fatty acid pattern score. (PDF)

S3 Text Cross-sectional analyses in EPIC-InterAct and US NHANES. (PDF)

## Abbreviations

ALT: alanine transaminase
AST: aspartate transaminase
BMI: body mass index
DNL: de novo lipogenesis
EPIC: European Prospective Investigation into Cancer and Nutrition
FA: fatty acid
FA-pattern score: fatty acid pattern score
GGT: γ-glutamyl transferase
HDL-C: high-density lipoprotein cholesterol
HR: hazard ratio
hsCRP: high-sensitivity C-reactive protein
MUFA: monounsaturated fatty acid
NHANES: National Health and Nutrition Examination Survey
PCA: principal component analysis
PUFA: polyunsaturated fatty acid
SFA: saturated fatty acid
T2D: type 2 diabetes
VLSFA: very-long-chain saturated fatty acid

## References

[1] Fessler MB, Rudel LL, Brown JM. Toll-like receptor signaling links dietary fatty acids to the metabolic syndrome. Curr Opin Lipidol. 2009;20(5):379–85. doi: 10.1097/MOL.0b013e32832fa5c4 19625959 10.1097/MOL.0b013e32832fa5c4 PMC3099529

[2] Jump DB, Tripathy S, Depner CM. fatty acid–regulated transcription factors in the liver. Annu Rev Nutr. 2013;33(1):249–69. doi: 10.1146/annurev-nutr-071812-161139 23528177 10.1146/annurev-nutr-071812-161139 PMC3940310

[3] Hodson L, Skeaff CM, Fielding BA. Fatty acid composition of adipose tissue and blood in humans and its use as a biomarker of dietary intake. Prog Lipid Res. 2008;47(5):348–80. doi: 10.1016/j.plipres.2008.03.003 18435934 10.1016/j.plipres.2008.03.003

[4] Baylin A, Campos H. The use of fatty acid biomarkers to reflect dietary intake. Curr Opin Lipidol. 2006;17(1):22–7. 16407712 10.1097/01.mol.0000199814.46720.83

[5] Wahli W, Michalik L. PPARs at the crossroads of lipid signaling and inflammation. Trends Endocrinol Metab. 2012;23(7):351–63. doi: 10.1016/j.tem.2012.05.001 22704720 10.1016/j.tem.2012.05.001

[6] Mozaffarian D, Wu JHY. Omega-3 fatty acids and cardiovascular disease: effects on risk factors, molecular pathways, and clinical events. J Am Coll Cardiol. 2011;58(20):2047–67. doi: 10.1016/j.jacc.2011.06.063 22051327 10.1016/j.jacc.2011.06.063

[7] Borkman M, Storlien LH, Pan DA, Jenkins AB, Chisholm DJ, Campbell L V. The relation between insulin sensitivity and the fatty-acid composition of skeletal-muscle phospholipids. N Engl J Med. 1993;328(4):238–44. doi: 10.1056/NEJM199301283280404 8418404 10.1056/NEJM199301283280404

[8] Poitout V, Robertson RP. Glucolipotoxicity: fuel excess and β-cell dysfunction. Endocr Rev. 2008;29(3):351–66. doi: 10.1210/er.2007-0023 18048763 10.1210/er.2007-0023 PMC2528858

[9] Rhee EP, Cheng S, Larson MG, Walford GA, Lewis GD, McCabe E, et al Lipid profiling identifies a triacylglycerol signature of insulin resistance and improves diabetes prediction in humans. J Clin Invest. 2011;121(4):1402–11. doi: 10.1172/JCI44442 21403394 10.1172/JCI44442 PMC3069773

[10] Vessby B, Lithell H. Interruption of long-term lipid-lowering treatment with bezafibrate in hypertriglyceridaemic patients. Effects on lipoprotein composition, lipase activities and the plasma lipid fatty acid spectrum. Atherosclerosis. 1990;82(1–2):137–43. doi: 10.1016/0021-9150(90)90152-9 2360915 10.1016/0021-9150(90)90152-9

[11] King IB, Lemaitre RN, Kestin M. Effect of a low-fat diet on fatty acid composition in red cells, plasma phospholipids, and cholesterol esters: investigation of a biomarker of total fat intake. Am J Clin Nutr. 2006;83(2):227–36. 16469979 10.1093/ajcn/83.2.227

[12] Warensjö E, Sundström J, Lind L, Vessby B, Warensjo E, Sundstrom J. Factor analysis of fatty acids in serum lipids as a measure of dietary fat quality in relation to the metabolic syndrome in men. Am J Clin Nutr. 2006;84(2):442–8. 16895896 10.1093/ajcn/84.1.442

[13] Anderson SG, Sanders TAB, Cruickshank JK. Plasma fatty acid composition as a predictor of arterial stiffness and mortality. Hypertension. 2009;53(5):839–45. doi: 10.1161/HYPERTENSIONAHA.108.123885 19307467 10.1161/HYPERTENSIONAHA.108.123885

[14] Dahm CC, Gorst-Rasmussen A, Jakobsen MU, Schmidt EB, Tjønneland A, Sørensen TIA, et al Adipose tissue fatty acid patterns and changes in anthropometry: a cohort study. PLoS ONE. 2011;6(7):e22587 doi: 10.1371/journal.pone.0022587 21811635 10.1371/journal.pone.0022587 PMC3141072

[15] Imamura F, Lemaitre RN, King IB, Song X, Lichtenstein AH, Matthan NR, et al Novel circulating fatty acid patterns and risk of cardiovascular disease: the Cardiovascular Health Study. Am J Clin Nutr. 2012;96(6):1252–61. doi: 10.3945/ajcn.112.039990 23097270 10.3945/ajcn.112.039990 PMC3497922

[16] Forouhi NG, Koulman A, Sharp SJ, Imamura F, Kröger J, Schulze MB, et al Differences in the prospective association between individual plasma phospholipid saturated fatty acids and incident type 2 diabetes: the EPIC-InterAct case-cohort study. Lancet Diab Endocrinol. 2014;2(10):810–8. doi: 10.1016/S2213-8587(14)70146-9 25107467 10.1016/S2213-8587(14)70146-9 PMC4196248

[17] Forouhi NG, Imamura F, Sharp SJ, Koulman A, Schulze MB, Zheng J, et al Association of plasma phospholipid n-3 and n-6 polyunsaturated fatty acids with type 2 diabetes: the EPIC-InterAct case-cohort study. PLoS Med. 2016;13(7):e1002094 doi: 10.1371/journal.pmed.1002094 27434045 10.1371/journal.pmed.1002094 PMC4951144

[18] InterAct Consortium. Design and cohort description of the InterAct Project: an examination of the interaction of genetic and lifestyle factors on the incidence of type 2 diabetes in the EPIC Study. Diabetologia. 2011;54(9):2272–82. doi: 10.1007/s00125-011-2182-9 21717116 10.1007/s00125-011-2182-9 PMC4222062

[19] Prentice RL. A case-cohort design for epidemiologic cohort studies and disease prevention trials. Biometrika. 1986;73(1):1–11.

[20] Wang L, Summerhill K, Rodriguez-Canas C, Mather I, Patel P, Eiden M, et al Development and validation of a robust automated analysis of plasma phospholipid fatty acids for metabolic phenotyping of large epidemiological studies. Genome Med. 2013;5(4):39 doi: 10.1186/gm443 23618465 10.1186/gm443 PMC3706814

[21] Wareham NJ, Jakes RW, Rennie KL, Schuit J, Mitchell J, Hennings S, et al Validity and repeatability of a simple index derived from the short physical activity questionnaire used in the European Prospective Investigation into Cancer and Nutrition (EPIC) study. Public Health Nutr. 2003;6(4):407–13. doi: 10.1079/PHN2002439 12795830 10.1079/PHN2002439

[22] Margetts BM, Pietinen P. European Prospective Investigation into Cancer and Nutrition: validity studies on dietary assessment methods. Int J Epidemiol. 1997;26(Suppl 1):S1–5. 9126528 10.1093/ije/26.suppl_1.s1

[23] Slimani N, Deharveng G, Unwin I, Southgate DAT, Vignat J, Skeie G, et al The EPIC nutrient database project (ENDB): a first attempt to standardize nutrient databases across the 10 European countries participating in the EPIC study. Eur J Clin Nutr. 2007;61(9):1037–56. doi: 10.1038/sj.ejcn.1602679 17375121 10.1038/sj.ejcn.1602679

[24] Scott RA, Fall T, Pasko D, Barker A, Sharp SJ, Arriola L, et al Common genetic variants highlight the role of insulin resistance and body fat distribution in type 2 diabetes, independent of obesity. Diabetes. 2014;63(12):4378–87. doi: 10.2337/db14-0319 24947364 10.2337/db14-0319 PMC4241116

[25] Locke AE, Kahali B, Berndt SI, Justice AE, Pers TH, Day FR, et al Genetic studies of body mass index yield new insights for obesity biology. Nature. 2015;518(7538):197–206. doi: 10.1038/nature14177 25673413 10.1038/nature14177 PMC4382211

[26] Ehrenberg ASC. Some questions about factor analysis. Statistician. 1962;12(3):191–208.

[27] Vigneau E, Qannari EM. Clustering of variables around latent components. Commun Stat Simul Comput. 2003;32(4):1131–50. doi: 10.1081/SAC-120023882

[28] Riley RD, Higgins JPT, Deeks JJ. Interpretation of random effects meta-analyses. BMJ. 2011;342:d549 doi: 10.1136/bmj.d549 21310794 10.1136/bmj.d549

[29] Durrleman S, Simon R. Flexible regression models with cubic splines. Stat Med. 1989;8(5):551–61. 2657958 10.1002/sim.4780080504

[30] Sterne JA, White IR, Carlin JB, Spratt M, Royston P, Kenward MG, et al Multiple imputation for missing data in epidemiological and clinical research: potential and pitfalls. BMJ. 2009;338:b2393 doi: 10.1136/bmj.b2393 19564179 10.1136/bmj.b2393 PMC2714692

[31] Efron B, Tibshirani RJ. Cross-validation and other estimates of prediction error In: Efron B, Tibshirani RJ, editors. An introduction to the bootstrap. Boca Raton (Florida): Chapman & Hall/CRC, Taylor & Francix Group; 1994 pp. 237–57.

[32] Spiegelman D, Hertzmark E. Easy SAS calculations for risk or prevalence ratios and differences. Am J Epidemiol. 2005;162(3):199–200. doi: 10.1093/aje/kwi188 15987728 10.1093/aje/kwi188

[33] Prati D, Taioli E, Zanella A, Della Torre E, Butelli S, Del Vecchio E, et al Updated definitions of healthy ranges for serum alanine aminotransferase levels. Ann Intern Med. 2002;137(1):1–10. doi: 10.7326/0003-4819-137-1-200207020-00006 12093239 10.7326/0003-4819-137-1-200207020-00006

[34] Maldonado G, Greenland S. simulation study of confounder-selection strategies. Am J Epidemiol. 1993;138(11):923–36. 8256780 10.1093/oxfordjournals.aje.a116813

[35] Jakobsson A, Jörgensen JA, Jacobsson A. Differential regulation of fatty acid elongation enzymes in brown adipocytes implies a unique role for Elovl3 during increased fatty acid oxidation. Am J Physiol Endocrinol Metab. 2005;289(4):E517–26. doi: 10.1152/ajpendo.00045.2005 15855229 10.1152/ajpendo.00045.2005

[36] Brown JM, Chung S, Sawyer JK, Degirolamo C, Alger HM, Nguyen T, et al Inhibition of stearoyl-coenzyme A desaturase 1 dissociates insulin resistance and obesity from atherosclerosis. Circulation. 2008;118(14):1467–75. doi: 10.1161/CIRCULATIONAHA.108.793182 18794388 10.1161/CIRCULATIONAHA.108.793182 PMC2716169

[37] Kersten S, Seydoux J, Peters JM, Gonzalez FJ, Desvergne B, Wahli W. Peroxisome proliferator-activated receptor alpha mediates the adaptive response to fasting. J Clin Invest. 1999;103(11):1489–98. doi: 10.1172/JCI6223 10359558 10.1172/JCI6223 PMC408372

[38] Fu S, Watkins SM, Hotamisligil GS. The role of endoplasmic reticulum in hepatic lipid homeostasis and stress signaling. Cell Metab. 2012;15(5):623–34. doi: 10.1016/j.cmet.2012.03.007 22560215 10.1016/j.cmet.2012.03.007

[39] Sevastianova K, Santos A, Kotronen A, Hakkarainen A, Makkonen J, Silander K, et al Effect of short-term carbohydrate overfeeding and long-term weight loss on liver fat in overweight humans. Am J Clin Nutr. 2012;96(4):727–34. doi: 10.3945/ajcn.112.038695 22952180 10.3945/ajcn.112.038695

[40] Lotta LA, Sharp SJ, Burgess S, Perry JRB, Stewart ID, Willems SM, et al Association between low-density lipoprotein cholesterol–lowering genetic variants and risk of type 2 diabetes. JAMA. 2016;316(13):1383 doi: 10.1001/jama.2016.14568 27701660 10.1001/jama.2016.14568 PMC5386134

[41] Lemaitre RN, King IB, Rice K, McKnight B, Sotoodehnia N, Rea TD, et al Erythrocyte very long-chain saturated fatty acids associated with lower risk of incident sudden cardiac arrest. Prostaglandins Leukot Essent Fatty Acids. 2014;91(4):149–53. doi: 10.1016/j.plefa.2014.07.010 25107579 10.1016/j.plefa.2014.07.010 PMC4156887

[42] Krachler B, Norberg M, Eriksson JW, Hallmans G, Johansson I, Vessby B, et al Fatty acid profile of the erythrocyte membrane preceding development of type 2 diabetes mellitus. Nutr Metab Cardiovasc Dis. 2008;18(7):503–10. doi: 10.1016/j.numecd.2007.04.005 18042359 10.1016/j.numecd.2007.04.005

[43] Grösch S, Schiffmann S, Geisslinger G. Chain length-specific properties of ceramides. Prog Lipid Res. 2012;51(1):50–62. doi: 10.1016/j.plipres.2011.11.001 22133871 10.1016/j.plipres.2011.11.001

[44] Abdullah MMH, Cyr A, Lépine M-C, Labonté M-È, Couture P, Jones PJH, et al Recommended dairy product intake modulates circulating fatty acid profile in healthy adults: a multi-centre cross-over study. Br J Nutr. 2015;113(3):435–44. doi: 10.1017/S0007114514003894 25609231 10.1017/S0007114514003894

[45] Weitkunat K, Schumann S, Nickel D, Hornemann S, Petzke KJ, Schulze MB, et al Odd-chain fatty acids as a biomarker for dietary fiber intake: a novel pathway for endogenous production from propionate. Am J Clin Nutr. 2017;105(6):1544–51. doi: 10.3945/ajcn.117.152702 28424190 10.3945/ajcn.117.152702

[46] Jenkins BJ, Seyssel K, Chiu S, Pan P-H, Lin S-Y, Stanley E, et al Odd chain fatty acids; new insights of the relationship between the gut microbiota, dietary intake, biosynthesis and glucose intolerance. Sci Rep. 2017;7:44845 doi: 10.1038/srep44845 28332596 10.1038/srep44845 PMC5362956

[47] Corrao G, Bagnardi V, Zambon A, La Vecchia C. A meta-analysis of alcohol consumption and the risk of 15 diseases. Prev Med. 2004;38(5):613–9. doi: 10.1016/j.ypmed.2003.11.027 15066364 10.1016/j.ypmed.2003.11.027

[48] Murase T, Misawa K, Minegishi Y, Aoki M, Ominami H, Suzuki Y, et al Coffee polyphenols suppress diet-induced body fat accumulation by downregulating SREBP-1c and related molecules in C57BL/6J mice. Am J Physiol Endocrinol Metab. 2011;300(1):E122–33. doi: 10.1152/ajpendo.00441.2010 20943752 10.1152/ajpendo.00441.2010

[49] Ding M, Bhupathiraju SN, Chen M, van Dam RM, Hu FB. Caffeinated and decaffeinated coffee consumption and risk of type 2 diabetes: a systematic review and a dose-response meta-analysis. Diabetes Care. 2014;37(2):569–86. doi: 10.2337/dc13-1203 24459154 10.2337/dc13-1203 PMC3898757

[50] Jacobs S, Kroger J, Floegel A, Boeing H, Drogan D, Pischon T, et al Evaluation of various biomarkers as potential mediators of the association between coffee consumption and incident type 2 diabetes in the EPIC-Potsdam Study. Am J Clin Nutr. 2014;100(3):891–900. doi: 10.3945/ajcn.113.080317 25057154 10.3945/ajcn.113.080317

[51] Vinknes KJ, Elshorbagy AK, Nurk E, Drevon CA, Gjesdal CG, Tell GS, et al Plasma stearoyl-CoA desaturase indices: association with lifestyle, diet, and body composition. Obesity. 2013;21(3):E294–302. doi: 10.1002/oby.20011 23404690 10.1002/oby.20011

[52] Imamura F, Micha R, Wu JHY, de Oliveira Otto MC, Otite FO, Abioye AI, et al Effects of saturated fat, polyunsaturated fat, monounsaturated fat, and carbohydrate on glucose-insulin homeostasis: a systematic review and meta-analysis of randomised controlled feeding trials. PLoS Med. 2016;13(7):e1002087 doi: 10.1371/journal.pmed.1002087 27434027 10.1371/journal.pmed.1002087 PMC4951141

[53] Barclay AW, Petocz P, McMillan-Price J, Flood VM, Prvan T, Mitchell P, et al Glycemic index, glycemic load, and chronic disease risk—a meta-analysis of observational studies. Am J Clin Nutr. 2008;87(3):627–37. 18326601 10.1093/ajcn/87.3.627

