# Supplementary material for: A combination of plasma phospholipid fatty acids and its association with incidence of type 2 diabetes: The EPIC-InterAct case-cohort study
Source: PLoS Med. 2017 Oct 11;14(10):e1002409. doi: 10.1371/journal.pmed.1002409 (PMC5636062; doi:10.1371/journal.pmed.1002409)
Supplement: S1 STROBE Checklist — (PDF) [file pmed.1002409.s001.pdf]

STROBE Statement—Checklist of items that should be included in reports of *cohort studies*

	Item No	Recommendation	Authors' inputs
**Title and abstract**	1	(*a*) Indicate the study's design with a commonly used term in the title or the abstract	The case-cohort design is noted.
		(*b*) Provide in the abstract an informative and balanced summary of what was done and what was found	Provided as recommended in the Abstract.
**Introduction**			
Background/rationale	2	Explain the scientific background and rationale for the investigation being reported	All the three paragraphs of the Introduction
Objectives	3	State specific objectives, including any prespecified hypotheses	The third paragraph of the Introduction
**Methods**			
Study design	4	Present key elements of study design early in the paper	The first paragraph of the Methods includes the study design. S1 Fig includes the flow diagram of the study design. S1 Text also includes additional information.
Setting	5	Describe the setting, locations, and relevant dates, including periods of recruitment, exposure, follow-up, and data collection	The first paragraph to the sixth paragraphs of the Methods inform those elements of this study. S1 Fig includes the flow diagram of the study design. S1 Text also includes additional information.
Participants	6	(*a*) Give the eligibility criteria, and the sources and methods of selection of participants. Describe methods of follow-up	The first paragraph of the Methods and S1 Fig clarify the information. The third paragraph of the Methods indicates the follow-up ascertainment of incident type 2 diabetes (outcome).
		(*b*) For matched studies, give matching criteria and number of exposed and unexposed	
Variables	7	Clearly define all outcomes, exposures, predictors, potential confounders, and effect modifiers. Give diagnostic criteria, if applicable	The Methods section included the information: the third paragraph for the outcome; the fourth paragraph and S1 Text include the main exposure assessment (fatty acid measurements); the fifth and sixth paragraphs and S1 Text include covariate assessment; the seventh and eighth paragraphs include derivation of the main exposure variable (the fatty-acid pattern score).
Data sources/ measurement	8*	For each variable of interest, give sources of data and details of methods of assessment (measurement). Describe comparability of assessment methods if there is more than one group	The Methods section included the information: the third paragraph for the outcome; the fourth paragraph and S1 Text include the main exposure assessment (fatty acid measurements); the fifth and sixth paragraphs and S1 Text include covariate assessment; the seventh

			and eighth paragraphs include derivation of the main exposure variable (the fatty-acid pattern score).
Bias	9	Describe any efforts to address potential sources of bias	P7-10 include documentation of adjustment for potential confounders and a number of sensitivity analyses. Effort was undertaken to minimise false positive findings in the cross-sectional analyses of dietary and metabolic factors, using data from the US national survey.
Study size	10	Explain how the study size was arrived at	The second paragraph of the Methods and S1 Fig inform how the final sample size was derived.
Quantitative variables	11	Explain how quantitative variables were handled in the analyses. If applicable, describe which groupings were chosen and why	The seventh and eighth paragraphs of the Methods include derivation of the main exposure variable (the fatty-acid pattern score) from fatty acid variables. The Derivation of genetic covariates is written in S1 Text. Other variables did not require special treatment. For the analysis of external validation, S2 Text includes the approach to deriving the main exposure variable.
Statistical methods	12	(*a*) Describe all statistical methods, including those used to control for confounding	The first and second paragraph of the Statistical analysis section include the information. S2 and S3 text also include additional statistical analyses.
		(*b*) Describe any methods used to examine subgroups and interactions	Noting a few post hoc analyses, the third paragraph of the Statistical analysis section includes the information.
		(*c*) Explain how missing data were addressed	The third paragraph of the statistical analysis section includes the information.
		(*d*) If applicable, explain how loss to follow-up was addressed	Not specifically explained in this paper. Loss to follow-up due to deaths was accounted for according to available data. Other loss such as moving away across regions was not captured. We observed no strong indication of substantial bias.
		(*e*) Describe any sensitivity analyses	The fourth paragraph of the Methods include the sensitivity analysis method for the main associations.
**Results**			
Participants	13*	(a) Report numbers of individuals at each stage of study—eg numbers potentially eligible, examined for eligibility, confirmed eligible, included in the study,	S1 Fig includes the information.

		completing follow-up, and analysed	
		(b) Give reasons for non-participation at each stage	Documented as the method to define a study population (the second paragraph of the Methods).S1 Fig includes the information as a flow diagram.
		(c) Consider use of a flow diagram	S1 Fig displays the flow diagram for this study
Descriptive data	14*	(a) Give characteristics of study participants (eg demographic, clinical, social) and information on exposures and potential confounders	S1 Table presents the information.
		(b) Indicate number of participants with missing data for each variable of interest	S2 Table presents the information.
		(c) Summarise follow-up time (eg, average and total amount)	The third paragraph of the Results (underneath "Associations with incidence of type 2 diabetes") includes the information of follow-up time.
Outcome data	15*	Report numbers of outcome events or summary measures over time	The third paragraph of the Results (underneath "Associations with incidence of type 2 diabetes") includes the information of follow-up time.
Main results	16	(*a*) Give unadjusted estimates and, if applicable, confounder-adjusted estimates and their precision (eg, 95% confidence interval). Make clear which confounders were adjusted for and why they were included	Table 2 includes the information.
		(*b*) Report category boundaries when continuous variables were categorized	As the main exposure variable (the fatty acid pattern score) has no unit, boundaries to split the study populations into 5 groups are not much informative (not of clinical utility) and thus not presented (without it Table 1 is sufficiently clear, we think).
		(*c*) If relevant, consider translating estimates of relative risk into absolute risk for a meaningful time period	For aetiological purpose of this work, the measure relevant to public health was cut for simplicity. That will be incorporated to the manuscript upon request: rate difference=-205 (-260, -150) per 100,000 according to the comparison of the 90th percentile to the 10th percentile value of the fatty acid pattern score.
Other analyses	17	Report other analyses done—eg analyses of subgroups and interactions, and sensitivity analyses	Figure 3, S2 Fig, S3 Table, S4 Table, S3 Fig are produced to reduce the concerns of internal validity and external validity of the main findings.
**Discussion**			
Key results	18	Summarise key results with reference to study objectives	The first paragraph of the Discussion.

Limitations	19	Discuss limitations of the study, taking into account sources of potential bias or imprecision. Discuss both direction and magnitude of any potential bias	The second last of this manuscript (counted before the Acknowledgement)
Interpretation	20	Give a cautious overall interpretation of results considering objectives, limitations, multiplicity of analyses, results from similar studies, and other relevant evidence	This study is unlikely to pose an issue of multiplicity in the primary single hypothesis tested, thus not explicitly discussed. No previous study was published to test the same hypothesis (see -14 include published studies using a similar method on different topics.
Generalisability	21	Discuss the generalisability (external validity) of the study results	The second last of this manuscript (counted before the Acknowledgement)
**Other information**			
Funding	22	Give the source of funding and the role of the funders for the present study and, if applicable, for the original study on which the present article is based	The second last of this manuscript (counted before the Acknowledgement)

*Give information separately for exposed and unexposed groups.

Note: An Explanation and Elaboration article discusses each checklist item and gives methodological background and published examples of transparent reporting. The STROBE checklist is best used in conjunction with this article (freely available on the Web sites of PLoS Medicine at http://www.plosmedicine.org/, Annals of Internal Medicine at http://www.annals.org/, and Epidemiology at http://www.epidem.com/). Information on the STROBE Initiative is available at http://www.strobe-statement.org.
