# Supplementary material for: A combination of plasma phospholipid fatty acids and its association with incidence of type 2 diabetes: The EPIC-InterAct case-cohort study
Source: PLoS Med. 2017 Oct 11;14(10):e1002409. doi: 10.1371/journal.pmed.1002409 (PMC5636062; doi:10.1371/journal.pmed.1002409)
Supplement: S1 Fig — (PDF) [file pmed.1002409.s002.pdf]

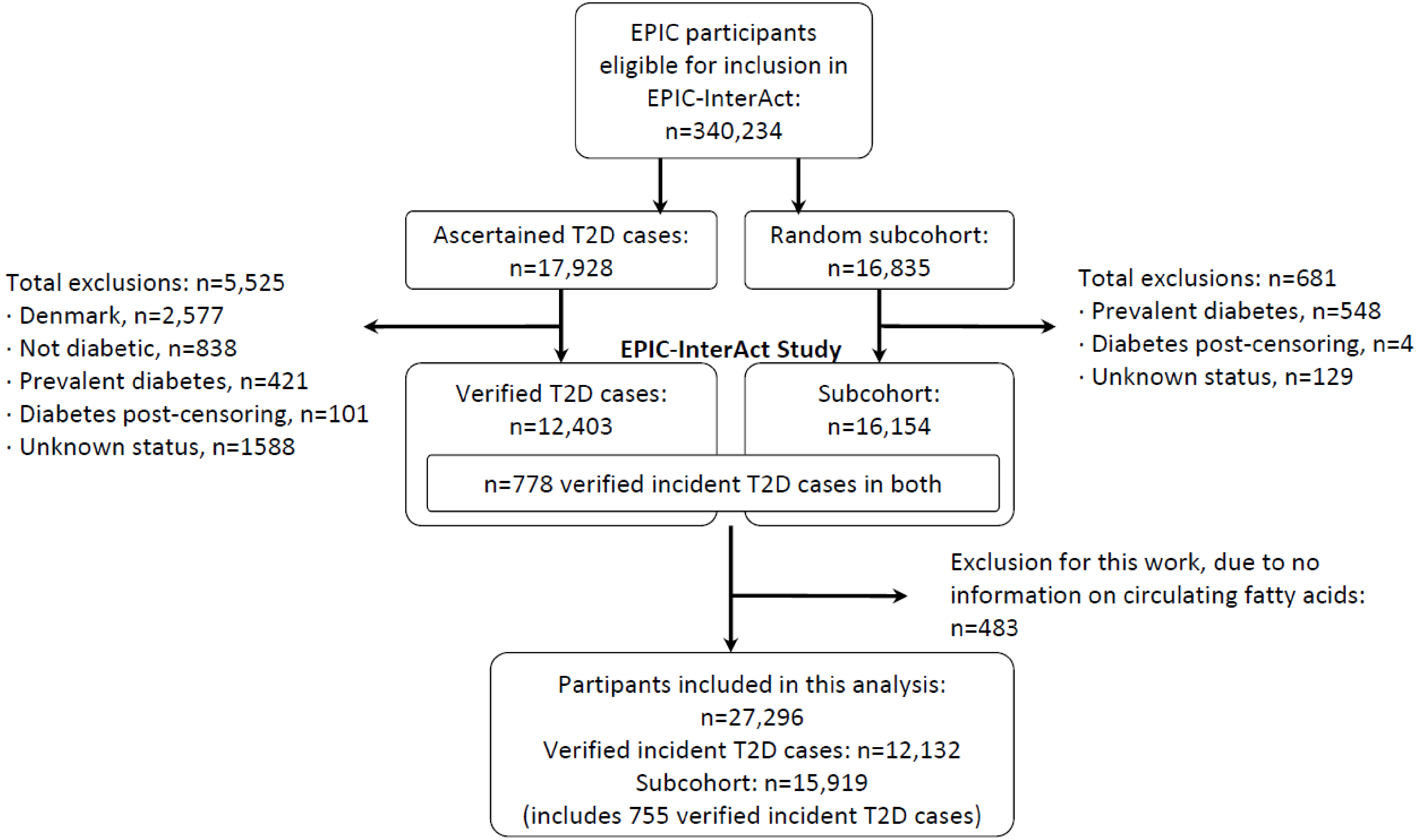

S1 Fig. Case-cohort study design of EPIC-InterAct and the selection of participants for the current analysis
