# Supplementary material for: A combination of plasma phospholipid fatty acids and its association with incidence of type 2 diabetes: The EPIC-InterAct case-cohort study
Source: PLoS Med. 2017 Oct 11;14(10):e1002409. doi: 10.1371/journal.pmed.1002409 (PMC5636062; doi:10.1371/journal.pmed.1002409)
Supplement: S2 Fig — (PDF) [file pmed.1002409.s003.pdf]

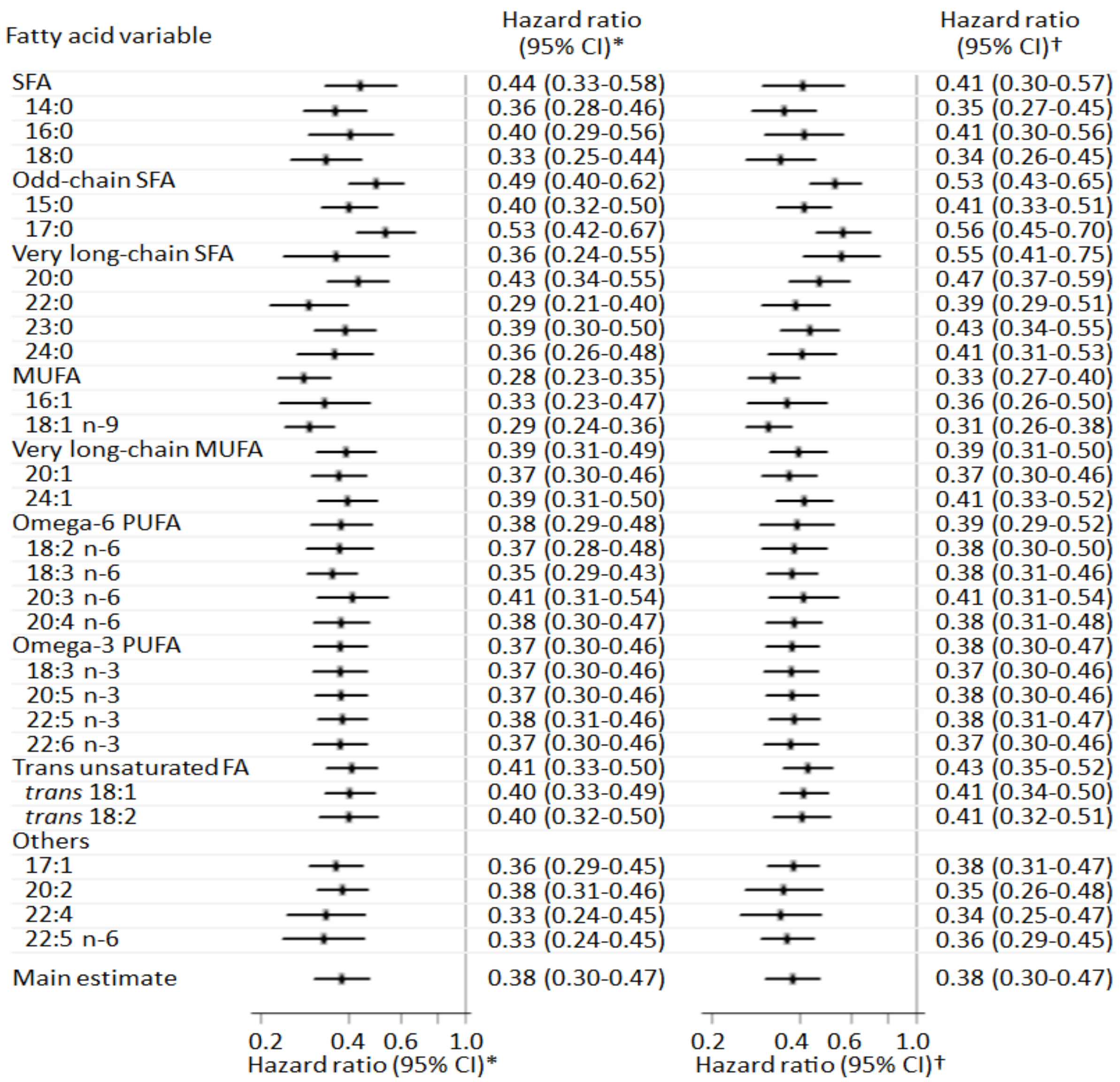

S2 Fig. Association of the fatty acid pattern score with incident type 2 diabetes: sensitivity analysis to examine influence of a single fatty acid or single fatty acid subclass. The hazard ratios (95% confidence interval, CI) on the left (*) were estimates adjusted for individual fatty acid (FA) or FA classes of each row. Those on the right (†) were estimates based on a FA pattern score derived from PCA not using one of single FA or FA subclasses of each row, where excluded FA variable was used as covariate for statistical adjustment. The estimate at the bottom was the main estimate based on the first fatty acid principal component. Analyses were performed among 27,296 adults in the EPIC-InterAct study. All hazard ratios were per interdecile range, estimated using the model same as the most adjusted model in Table 2.

Abbreviations: CI, confidence interval; FA, fatty acids; MUFA, monounsaturated fatty acids; PUFA, polyunsaturated fatty acids; SFA, saturated fatty acids.
