# Supplementary material for: A combination of plasma phospholipid fatty acids and its association with incidence of type 2 diabetes: The EPIC-InterAct case-cohort study
Source: PLoS Med. 2017 Oct 11;14(10):e1002409. doi: 10.1371/journal.pmed.1002409 (PMC5636062; doi:10.1371/journal.pmed.1002409)
Supplement: S3 Fig — (PDF) [file pmed.1002409.s004.pdf]

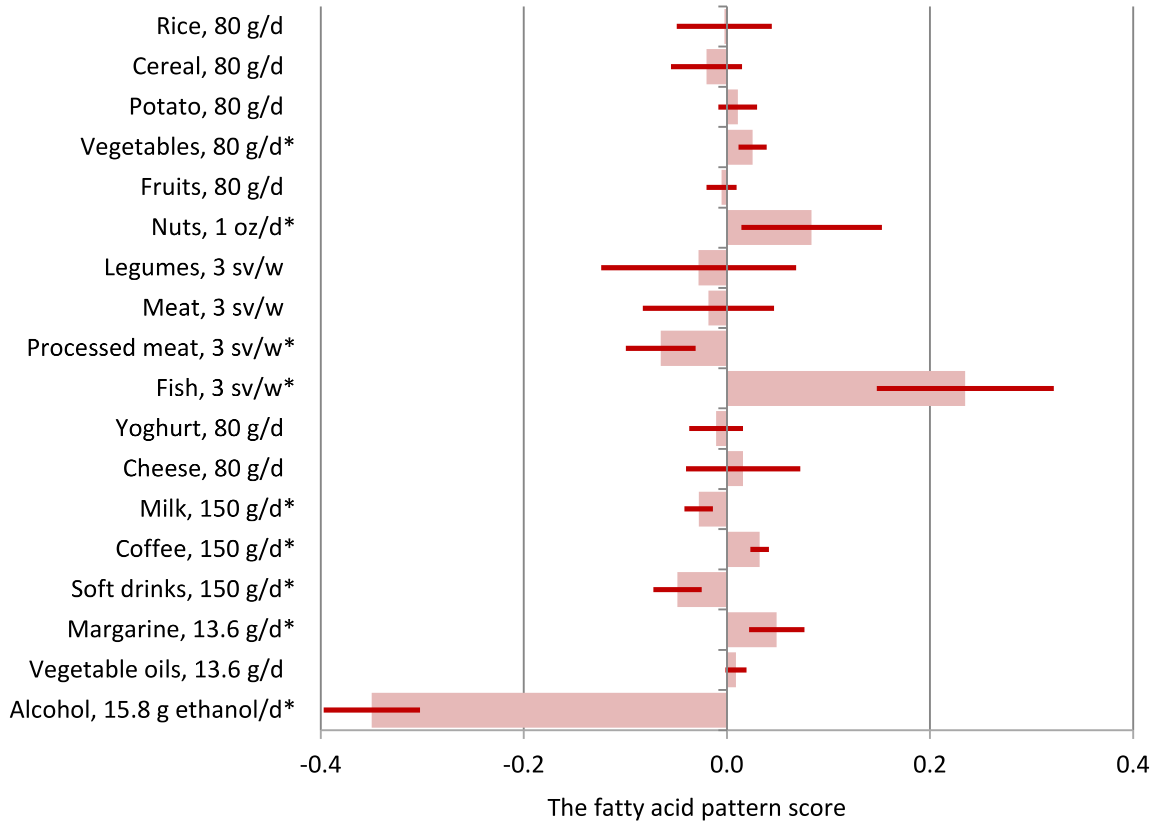

S3 Fig. Cross-sectional association between dietary consumption of major food groups and the main fatty acid pattern score: EPIC-InterAct (n=15,566). Bars represent linear associations of dietary factors with the fatty acid pattern score (scaled per one standard deviation); error bars, 95% confidence interval. Linear regression models were fitted, including the fatty acid score (standardized within each country) as a dependent variable and each of the dietary factors listed on the left as a main independent variable. The model adjusted for potential confounders as modelled for analysis of Table 2. Alcohol consumption was separately examined. The other dietary factors listed were evaluated separately, with adjustment for alcohol consumption (continuous). Estimates were scaled as presented in the left column (sv, serving; oz, ounce defined as 28.8 g).

* Statistically significant association ($p<0.05$), without adjusted for other variables. Mutual adjustment was performed in the main analysis (Figure 2 of the main document) to identify independent dietary determinants for the main fatty acid pattern was performed and test the external validity (see supplementary text for details).
