# Supplementary material for: A combination of plasma phospholipid fatty acids and its association with incidence of type 2 diabetes: The EPIC-InterAct case-cohort study
Source: PLoS Med. 2017 Oct 11;14(10):e1002409. doi: 10.1371/journal.pmed.1002409 (PMC5636062; doi:10.1371/journal.pmed.1002409)
Supplement: S1 Protocol — (PDF) [file pmed.1002409.s005.pdf]

Protocol: A combination of plasma phospholipid fatty acids and incidence of type 2 diabetes in EPIC-InterAct

Proposer: Fumiaki Imamura, Nita Forouhi
Affiliation: MRC Epidemiology Unit

Working group participants: Stephen J. Sharp, Albert Koulman, Matthias B. Schulze, Janine Kröger, Julian L. Griffin, José María Huerta, Marcela Guevara: See progress of the ongoing parental project: “The association between plasma phospholipid fatty acids and incident type 2 diabetes in European populations: EPIC-InterAct study” (publication ID=212) (see Appendix of this document).

Research question: Fatty acids are likely to reflect our metabolic state and environmental exposure jointly by combination. For example, polyunsaturated fatty acids can serve as ligands of nuclear receptors (e.g. PPARs) which alter activities of synthesis of fatty acids in the liver and other tissues. Also, dietary intakes such as alcohol and sugars stimulate synthesis of multiple fatty acids and suppress fatty acid oxidation, which influence multiple fatty acids simultaneously. Thus, a combination of fatty acids may have greater biological implication than single individual fatty acids (e.g. 16:0) or fatty acid subclasses (e.g. long-chain saturated fatty acids). This potential implication has been examined previously for cardiovascular disease outcomes [1] but not yet for diabetes. Therefore, in addition to the ongoing project of assessments of individual fatty acids for each association with incident type 2 diabetes, we design this exploratory analysis to capture a pattern of fatty acids and examine its association with incidence of type 2 diabetes and related traits.

Objectives
(1) To identify a major fatty acid pattern using principal component analysis.
(2) To estimate the prospective association between the score representing the major fatty acid pattern and incidence of type 2 diabetes.
(3) To characterise the cross-sectional association between the score and metabolic and dietary factors.

Main exposures: the fatty acid pattern score derived from individual fatty acids. Individual fatty acids with a mean concentration < 0.05% will be filtered out, following the main project of individual fatty acids.

Main potential confounding factors: not pre-specified specifically, to select with considerations of biological plausibility, influences on the main parameter estimate, and parsimony of models. Covariates include centre, age, sex, total energy intake, carbohydrate intake, protein intake, alcohol intake, smoking, education, physical activity, family history of diabetes, prevalent clinical conditions (dyslipidaemia, hypertension, coronary heart disease, stroke, cancer, diabetes), body mass index, waist circumference, dietary intakes, and conventional risk factors for cardiometabolic diseases (e.g. lipid, C-reactive protein).

Analysis Plan
Exclusions: Participants with prevalent diabetes, without fatty acid measures, or without information on incident type 2 diabetes.

Pattern derivation: Principal component analysis to identify a major fatty acid pattern (the first principal component), weighted to account for the case-cohort design from the multiple cohorts.

Up on the prior experience [1], an identification of one component is feasible, interpretable, and acceptable to allow thorough analysis to advance biological understanding.

Statistical analysis:
To follow general procedures in ongoing InterAct analysis (see the original plan for individual fatty acids) and account for prior analysis or concerns [1,2], including pattern description, covariate imputation and sensitivity analyses.

- ➔ Based on comparison between results from single and multiple imputation, single imputation is used throughout
- ➔ Sensitivity analysis to compare between single imputation, multiple imputation and complete-case analysis

Prospective associations of the fatty acid pattern score with type 2 diabetes: Prentice-weighted Cox regression, with adjustment for potential confounders, fitting models within each country and combining across countries using random effects meta-analysis. Test for interaction include age, sex, BMI, but if with further reading of the literature, other factors emerge to be important, we will include these at analysis stage.

Covariate selection: Adiposity (body mass index) and circulating lipids are likely to alter the findings because they are on the pathway of fatty acid synthesis as drivers or consequences. Accounting for such biological plausibility, the most clinically meaningful results from multiple multivariable-adjusted models.

Sensitivity analyses: to address the general concerns of pattern recognition approach [2] as well as those planned for the analyses of individual fatty acids:

- Stratification by risk set determined by a duration of follow-up
- Comparison between imputation analysis and complete-case analysis
- Split-sample two-stage analysis (doing PCA in the subset, and testing the association in the rest) (to be done by treating countries as subsets and other main characteristics: age, sex and BMI).
- Adjustment for components of PCA (individual fatty acids and fatty acid subclasses) to rule out that an observed association would be driven by a single factor

Cross-sectional associations of the fatty acid pattern score with metabolic factors, including adiposity measures, circulating lipids, inflammatory cytokines, and hepatic markers.
Cross-sectional associations of the fatty acid pattern score with dietary factors, including the food groups as listed in the Analysis Plan for individual fatty acids; and macronutrient distribution (% of energy from available macronutrient variables)

AMMENDMENT:
May 2014:
NHANES data appear to be available: https://wwwn.cdc.gov/nchs/nhanes/2003-2004/SSFA_C.htm . Cross-sectional analyses for external validation of associations of the fatty acid pattern with metabolic and dietary factors are to be done, following the approach previously conducted [1].

August 2016:
Genetic data are added. In the InterAct meeting on 2 October 2015 at Clare College, a suggestion was given to examine selected genetic risk scores as potential determinants of the fatty acid pattern in the InterAct. See data-release information (PHIA0000292016_IA44_02Aug).

References:

1. Imamura F, Lemaitre RN, King IB, Song X, Lichtenstein AH, Matthan NR, et al. Novel circulating fatty acid patterns and risk of cardiovascular disease: the Cardiovascular Health Study. Am J Clin Nutr. 2012;96(6):1252–61. doi:10.3945/ajcn.112.039990
2. Imamura F, Jacques PF. Invited commentary: dietary pattern analysis. Am J Epidemiol. 2011;173(10):1105–8. doi:10.1093/aje/kwr063

Appendix

The association between plasma phospholipid fatty acids and incident type 2 diabetes in European populations: EPIC-InterAct study

Proposer: Nita Forouhi
Affiliation: MRC Epidemiology Unit

Already identified working group participants: Stephen Sharp, Albert Koulman

Research question: The evidence for the contribution of the amount and type of dietary fat intake to the development of diabetes is inconsistent. Saturated fatty acid intake has long been proposed to be a risk factor for insulin resistance and diabetes mellitus, but review of the available evidence indicates surprisingly equivocal findings from both controlled trials and prospective cohort studies. For other classes of fatty acids there are also similarly equivocal findings. For instance, while it is proposed that long-chain fatty acids of the n-3 class, such as eicosapentaenoic acid (EPA) and docosahexaenoic acid (DHA) will be inversely associated with diabetes risk, the converse has been shown with increased risk of diabetes with dietary intake of these fatty acids. A common feature of research to date is the estimation of fatty acids from self-report dietary questionnaires which are prone to measurement error. Fatty acids in blood fractions can be used as objective markers of dietary fat intake, and offer more precise measurement. We now have the opportunity to examine these associations using objectively measured fatty acids in the plasma phospholipid fraction in EPIC-InterAct.

Objectives
(1) To describe the distribution of plasma fatty acids (P-FA) in the subcohort, including distribution by country, age, sex, BMI.
(2) To describe the associations between P-FA and food intake from FFQs, using the subcohort.
(3) To estimate the association between P-FA profiles and incident T2D, and to compare these with estimates obtained using classes of fatty acids derived from FFQs.

Main exposures: 38 P-FA; P-FA classes: SFA, n-3 PUFA, n-6 PUFA, MUFA, trans-FA.
FFQ foods:
1. Total meat, red meat, processed meat, offals, white meat.
2. Total dairy products, milk, yoghurt, thick fermented milk, cheese, butter, other dairy.
3. Total fish, lean fish, fatty fish, shellfish.
4. Other fat-rich foods such as nuts and seeds, cakes, oils.

Main potential confounding factors: Centre, age, sex, BMI, WC, total energy intake, carbohydrate intake, protein intake, alcohol intake, smoking, education, physical activity, family history of diabetes, prevalent chronic disease (MI, stroke, cancer, diabetes), fibre intake, sugar sweetened beverages, coffee intake, HbA1c.

Other specific analytical issues: Correlation coefficients will be calculated for each food/fatty acid or fatty acid combinations. Associations will be estimated using Prentice-weighted Cox regression, with adjustment for potential confounders. Models will be fit separately within each country and combined across countries using random effects meta-analysis. We will examine these effects with the exposure as a continuous variable (per SD change) as well as in quantiles (e.g. tertiles). We will test for interaction between each main class of fatty acid and (1) age, (2) BMI, but if with further reading of the literature, other factors emerge to be important, we will include these at analysis stage.

Analysis Plan
Exclusions: Individual fatty acids with a mean concentration < 0.05% will not be analysed.

Descriptive statistics: The number of individuals with fatty acid measurements will be presented separately by country and case/subcohort status. Using data from the subcohort, the mean and SD of each fatty acid and fatty acid class will be presented separately by country, age (<40, 40-<60, ?60 years), sex and BMI (<25, 25-<30, ?30 kg/m2). Correlations between fatty acid classes measured in plasma and derived from FFQ in the subcohort.

Spearman rank correlation coefficients will be calculated between each class of fatty acid measured in plasma and derived from FFQ, within the subcohort. For each pair of measures, a p-value testing the null hypothesis that the true correlation is 0 will be calculated.

Associations between fatty acids measured in plasma or derived from FFQ and food intakes assessed by FFQ in the subcohort. Spearman rank correlation coefficients will be calculated between each plasma fatty acid (individual and classes) and the following food intake variables from FFQ, within the subcohort:

1. Red meat, processed meat, red and processed meat, poultry.
2. Total dairy, milk, yoghurt/thick fermented milk, cheese, butter.
3. Fish and shellfish, lean fish, fatty/very fatty fish.
4. Vegetable oils, olive oil, margarine, cakes and cookies, nuts and seeds.
5. Soft drinks, alcohol.
6. Cereal and cereal products, pasta/rice, bread, potatoes

We will also examine correlations between P-FAs and macronutrients (carbohydrate intake, protein intake, fat intake). Correlation coefficients will also be calculated between each class of fatty acid derived from FFQ (i.e. total fat, total SFA, PUFA, MUFA) and each of the food intake variables listed above. For each pair of measures, a p-value testing the null hypothesis that the true correlation is 0 will be calculated.

Associations between P-FA and incident T2D: The hazard ratios and 95% CIs will be estimated for each individual fatty acid and also for each class of fatty acid (i.e. SFA, n-3 PUFA, n-6 PUFA, MUFA, trans-FA). SDs and cut-offs for categorisation will be calculated using the data in the subcohort.

The following additional groupings of fatty acids will be included as exposures:
• ratio of n-6 to n-3 PUFA.
•estimation of desaturase activity using the following product to precursor ratios:
(1) 16:1n7 / 16:0;
(2) 18:1n9 / 18:0 to reflect SCD (stearoyl coenzyme A desaturase, or delta-9 desaturase) activity that catalyses the synthesis of MUFA from SFA;
(3) 18:3n6 / 18:2n6 to reflect delta-6 desaturase (D6D) activity;
(4) 20:4n6 / 20:3n6 to reflect delta-5 desaturase (D5D) activity required for synthesis of n-6 and n-3 PUFA.
•individual fatty acids will be summed where the concentrations of each individual fatty acid are low and the combination is appropriate as informed by the literature (below list is not exhaustive):
(1) C15:0 + C17:0 to reflect SFA from dairy products.
(2) EPA+DHA+DPA (and EPA+DHA) to reflect n-3 PUFA of marine origin

Hazard ratios and 95% CIs will be estimated using Prentice-weighted Cox regression models, fit separately within each country and combined across countries using random effects meta-analysis. To adjust for potential confounders, 3 models will be fit for each fatty acid exposure within each country:

Model 1 – adjusted for centre, sex, physical activity, smoking, education level.
Model 2 – as for model 1, plus total energy intake, alcohol.
Model 3 – as for model 2, plus BMI.

Hazard ratios and 95% CIs will be estimated for each class of fatty acid derived from FFQ (i.e. total fat, total SFA, PUFA, MUFA), using the same exposure definitions and models described above.
