# Supplementary material for: A combination of plasma phospholipid fatty acids and its association with incidence of type 2 diabetes: The EPIC-InterAct case-cohort study
Source: PLoS Med. 2017 Oct 11;14(10):e1002409. doi: 10.1371/journal.pmed.1002409 (PMC5636062; doi:10.1371/journal.pmed.1002409)
Supplement: S1 Table — (PDF) [file pmed.1002409.s006.pdf]

S1 Table. Baseline characteristics according to quintile categories of the fatty acid pattern score in the sub-cohort of EPIC-InterAct (n=15,919).*

	Quintile categories of the fatty acid pattern score*					
	I	II	III	IV	V	
Age, years	53 (9)	52 (9)	52 (9)	52 (9)	53 (9)	
Sex, % men	47	39	37	34	30	†
Education, ≥high school, %	36	36	38	36	34	
Current smokers, %	32	26	25	24	22	†
High physical activity, %	44	44	42	42	41	
Postmenopausal, % in women	28	29	30	33	38	
Hormone therapy, % in women	21	15	13	13	12	†
Family history of diabetes, % ‡	20	21	19	18	18	
Prevalent diseases						
Hypertension, %	24	21	17	17	14	†
Dyslipidaemia, %	26	19	17	15	14	†
Coronary heart disease, %	2.5	2.1	2.8	2.1	2.1	
Dietary consumption						
Energy, mega joules/day	9.3 (2.8)	9 (2.7)	8.9 (2.6)	8.8 (2.6)	8.8 (2.5)	†
Alcohol, g/day	20 (19)	12 (14)	9 (11)	7 (10)	6 (9)	†
Fibre, g/day	21 (6)	22 (6)	22 (6)	23 (6)	23 (6)	†
Vegetables, g/day	160 (101)	171 (109)	179 (113)	191 (125)	208 (128)	†
Fruits, g/day	216 (181)	229 (177)	238 (183)	237 (183)	243 (183)	†
Soft drinks, g/day	78 (184)	69 (149)	67 (150)	68 (145)	62 (138)	†
Processed meat, g/day	35 (28)	34 (27)	33 (26)	33 (25)	31 (24)	†
Body mass index, kg/m^2	26.7 (4.4)	26.2 (4.3)	26.0 (4.2)	25.6 (4.1)	25.5 (4.0)	†
Haemoglobin A1c, mmol/mol	36.2 (5.9)	36.2 (5.1)	36.2 (4.7)	36.2 (4.2)	36.3 (4.2)	
Triglycerides, mmol/L	1.8 (1.2)	1.4 (0.9)	1.3 (0.7)	1.2 (0.7)	1.1 (0.6)	†
High-density lipoprotein, mmol/L	1.47 (0.45)	1.49 (0.42)	1.48 (0.41)	1.51 (0.41)	1.53 (0.41)	†
C-reactive protein, μmol/L	2.6 (4.0)	2.3 (4.0)	2.1 (3.9)	1.9 (3.6)	2.0 (3.9)	†
Gamma glutamyltransferase, U/L	30 (37)	44 (60)	29 (30)	27 (30)	24 (25)	†
Genetic risk score, z-score						
Insulin resistance	0.03 (1.0)	-0.00 (1.0)	0.01 (1.0)	-0.02 (1.0)	-0.02 (1.0)	
Body-mass index	-0.02 (1.0)	0.01 (1.0)	0.01 (1.0)	-0.01 (1.0)	0.01 (1.0)	
Fatty acid classes, % total §						
Saturated FA (14:0+16:0+18:0)	46.3 (1)	46.1 (1.1)	45.9 (1.2)	45.8 (1.2)	45.8 (1.4)	†
Monounsaturated FA (16:1+18:1)	12.5 (2)	11.4 (1.6)	10.9 (1.6)	10.5 (1.5)	9.8 (1.7)	†
Omega-3 PUFA (EPA+DPA+DHA)	5.9 (1.6)	6.2 (1.8)	6.5 (1.9)	6.7 (2.0)	6.9 (2.2)	†
Trans FA (trans-18:1+trans-18:2)	0.2 (0.1)	0.3 (0.2)	0.3 (0.2)	0.4 (0.2)	0.4 (0.2)	†
Odd-chain saturated FA (15:0+17:0)	0.5 (0.1)	0.6 (0.1)	0.6 (0.1)	0.7 (0.1)	0.7 (0.1)	†

* EPIC-InterAct, European Prospective Investigation into Cancer and Nutrition-InterAct (EPIC-InterAct), using the fatty acid pattern score generated by applying principal component analysis to 27 individual fatty acids of circulating phospholipids. Values represent means (standard deviations) for continuous variables and percentages for categorical variables.

† *p* for trend<0.01 across quintile categories.

‡ not available in two research centres, thus evaluated among 11,111 adults.

§ % of total phospholipid fatty acids is presented. Each of 27 fatty acids is shown in Table 1.

Abbreviations: FA, fatty acids; PUFA, polyunsaturated fatty acids; EPA, eicosapentaenoic acid; DPA, docosapentaenoic acid; and DHA, docosahexaenoic acid.
