# Supplementary material for: A combination of plasma phospholipid fatty acids and its association with incidence of type 2 diabetes: The EPIC-InterAct case-cohort study
Source: PLoS Med. 2017 Oct 11;14(10):e1002409. doi: 10.1371/journal.pmed.1002409 (PMC5636062; doi:10.1371/journal.pmed.1002409)
Supplement: S2 Table — (PDF) [file pmed.1002409.s007.pdf]

S2 Table. Baseline characteristics according to presence or absence of missing information among participants with fatty acid measures: EPIC-InterAct (n=27,296).

	% missing	No missing n=23,039	1≥ missing* n=4,257	p-value
Age, years	0	53.6 (8.7)	54.3 (8.9)	0.006
Sex, % men	0	43	43	0.68
Education, ≥high school, %	2.1	32	30	<0.001
Smoking status, %	1.2			
Former smokers		29	30	
Current smokers		26	28	0.004
High physical activity, %	1.4	40	41	0.001
Postmenopausal, % in women	0	57	57	0.77
Hormone use, % in women	4.4	14	15	0.13
Family history of diabetes, %†	49.9	27	27	<0.001
Prevalent diseases				
Hypertension, %	2.7	27	28	0.95
Dyslipidaemia, %	4.2	26	24	0.01
Coronary heart disease, %‡	0	3.6	3.5	0.08
Dietary consumption				
Energy, mega joules	2.6	9.0 (2.7)	8.9 (2.8)	0.67
Alcohol, g	0.4	12 (15)	8.8 (13)	<0.001
Fibre, g	2.6	21.9 (5.9)	21.7 (6.1)	0.02
Vegetables, g	2.6	181 (118)	167 (112)	0.57
Fruits, g	2.6	232 (184)	212 (170)	0.02
Soft drinks, g	2.6	75 (167)	99 (195)	0.002
Processed meat, g	2.6	34.9 (27.7)	35.3 (26.3)	<0.001
Selected circulating fatty acids (FA) , % total fatty acids				
Saturated FA (16:0+18:0)	0	46.1 (1.2)	46.3 (1.2)	<0.001
Monounsaturated FA (16:1+18:1)	0	11.1 (1.9)	11.2 (1.8)	<0.001
Trans FA (trans 18:1+18:2)	0	0.29 (0.17)	0.36 (0.19)	<0.001
n-3 polyunsaturated FA	0	6.4 (1.9)	6.7 (2.0)	<0.001
n-6 polyunsaturated FA	0	31.6 (3.0)	31.0 (2.9)	0.05
Body mass index, kg/m^2	0.7	27.6 (4.8)	27.7 (5.1)	<0.001
Triglycerides, mmol/L	4.6	1.60 (1.18)	1.69 (1.18)	0.26
High-density lipoprotein, mmol/L	4.6	1.40 (0.42)	1.37 (0.42)	0.84
C-reactive protein, µmol/L	4.6	2.9 (4.9)	3.1 (5.1)	0.72
Fatty acid pattern score (scaled to mean=0, sd=1 of randomly-sampled population)	0	-0.23	-0.23	0.79
Follow-up period	0	9.8 (3.7)	9.7 (3.9)	
Incident diabetes cases, % (rate) among randomly-sampled participants	0	4.6 (384/100,000)	5.7 (476/100,000)	<0.001

* EPIC-InterAct, European Prospective Investigation into Cancer and Nutrition-InterAct. Participants missing one or more of the listed variables in the first column. P-values were obtained by multivariable logistic regression examining missing for 1 or more variable as a binary outcome (yes/no) and all the variables listed in the left column as predictors, except different subtypes of fatty acids. The fatty acid subtypes were examined simultaneously in a model not including the fatty acid pattern variable.

† Family history of diabetes was not assessed in centres in Italy and Spain or in 1 UK centre (Oxford) or 1 German centre (Heidelberg). In the other 6 countries, 24.5% of participants had the missing information. This variable was neither accounted in the subgroup with missing information nor as a confounder (tested as yes, no, or unknown) in main analyses.

‡ Coded as yes, if a prior history was self-reported.
