# Supplementary material for: A combination of plasma phospholipid fatty acids and its association with incidence of type 2 diabetes: The EPIC-InterAct case-cohort study
Source: PLoS Med. 2017 Oct 11;14(10):e1002409. doi: 10.1371/journal.pmed.1002409 (PMC5636062; doi:10.1371/journal.pmed.1002409)
Supplement: S3 Table — (PDF) [file pmed.1002409.s008.pdf]

S3 Table. Prospective associations of the fatty acid pattern score with incident type 2 diabetes in EPIC-InterAct: assessment of influence of subsets of covariates, missing information, duration of follow-up, and normality of fatty acid variables. *

	Pooled hazard ratio (95% confidence interval) per he 10th to 90th percentile range	
	adjusted for potential confounders	additionally adjusted for adiposity and lipids
Primary analysis	0.28 (0.24-0.32)	0.38 (0.30-0.47)
Adjusted for random glucose, C-reactive protein, hepatic enzymes	0.31 (0.27-0.37)	0.40 (0.32-0.51)
Adjusted for coffee, sugar-sweetened beverages, fish, and margarine (independent dietary correlates)	0.28 (0.24-0.32)	0.41 (0.33-0.50)
Adjusted for percentages of energy from each of total saturated fatty acids, total monounsaturated fatty acids, and total polyunsaturated fatty acids.	0.27 (0.25-0.30)	0.39 (0.31-0.48)
Adjusted for genetic scores representing insulin resistance and body-mass index	0.28 (0.24-0.33)	0.40 (0.32-0.49)
Complete-case analysis	0.28 (0.25-0.32)	0.38 (0.32-0.46)
Multiple imputation (20 imputed datasets)	0.29 (0.25-0.33)	0.39 (0.32-0.47)
Evaluated time-dependency in the associations		
0-7 years of follow-up	0.27 (0.24-0.30)	0.37 (0.30-0.44)
>7 years of follow-up	0.31 (0.25-0.37)	0.42 (0.34-0.52)
>2 years of follow-up	0.28 (0.24-0.33)	0.40 (0.32-0.51)
After improving normal distribution of 27 fatty acid variables	0.26 (0.22-0.31)	0.35 (0.28-0.44)

* All of these analyses evaluated 27,296 adults in the case-cohort sample in European Prospective Investigation into Cancer and Nutrition-InterAct study (EPIC-InterAct), except complete-case analysis (n=23,039). Multivariable-adjusted Prentice-weighted Cox regression models were fitted in each country of EPIC-InterAct; country-specific estimated hazard ratios were pooled using random-effects meta-analysis. Models included the same covariates as the first and third models presented in main Table 2, unless mentioned.
