# Supplementary material for: A combination of plasma phospholipid fatty acids and its association with incidence of type 2 diabetes: The EPIC-InterAct case-cohort study
Source: PLoS Med. 2017 Oct 11;14(10):e1002409. doi: 10.1371/journal.pmed.1002409 (PMC5636062; doi:10.1371/journal.pmed.1002409)
Supplement: S4 Table — (PDF) [file pmed.1002409.s009.pdf]

S4 Table. Internal cross-validation for derivation of the fatty acid pattern score and its prospective association with incident type 2 diabetes in EPIC-InterAct: sensitivity analysis to examine internal validity.*

Derivation set to derive a fatty acid pattern score		Validation set in which longitudinal analysis was performed, using the fatty acid pattern score calculated based on a pattern structure derived from the Derivation set.				
Population from the sub-cohort	n	Population	n	Correlation coefficient†	HR (95% CI) ‡ adjusted for potential confounders	HR (95% CI)‡ additionally adjusted for adiposity and lipids
All adults	15,919	All	27,296	1.0	0.28 (0.24-0.32)	0.38 (0.30-0.47)
Country						
France excluded	15,332	France	866	>0.99	0.18 (0.13-0.24)	0.23 (0.16-0.33)
Italy excluded	13,913	Italy	3,369	>0.99	0.38 (0.34-0.44)	0.50 (0.43-0.58)
Spain excluded	12,364	Spain	5,837	>0.99	0.41 (0.37-0.45)	0.59 (0.52-0.66)
United Kingdom excluded	14,609	United Kingdom	2,296	>0.99	0.25 (0.22-0.30)	0.28 (0.24-0.34)
Netherlands excluded	14,419	Netherlands	2,283	>0.99	0.26 (0.22-0.31)	0.34 (0.28-0.43)
Germany excluded	13,874	Germany	3,562	>0.99	0.36 (0.32-0.41)	0.54 (0.47-0.63)
Sweden excluded	13,093	Sweden	5,128	>0.99	0.32 (0.29-0.35)	0.43 (0.38-0.48)
Denmark excluded	2,826	Denmark	3,955	>0.99	0.32 (0.29-0.36)	0.43 (0.38-0.49)
		Pooled §	27,296	>0.99	0.31 (0.27-0.36)	0.40 (0.34-0.50)
Age						
≥55 years	6,386	<55 years	14,742	>0.99	0.23 (0.18-0.28)	0.32 (0.23-0.42)
<55 years	9,533	≥55 years	12,554	>0.99	0.30 (0.26-0.35)	0.39 (0.31-0.48)
		Pooled §	27,296	>0.99	0.34 (0.27-0.44)	0.42 (0.30-0.57)
Sex						
Women	9,917	Men	11,659	0.99	0.31 (0.25-0.39)	0.39 (0.32-0.47)
Men	6,002	Women	15,637	0.99	0.26 (0.21-0.30)	0.37 (0.29-0.48)
		Pooled §	27,296	0.99	0.26 (0.20-0.35)	0.36 (0.30-0.44)
Adiposity						
BMI≥25.0 kg/m^2	2,512	BMI<25.0 kg/m^2	19,804	>0.99	0.39 (0.34-0.44)	0.48 (0.41-0.56)
BMI<25.0 kg/m^2	13,307	BMI≥25.0 kg/m^2	7,295	>0.99	0.30 (0.25-0.36)	0.35 (0.27-0.45)
		Pooled §	27,296	>0.99	0.28 (0.23-0.34)	0.38 (0.33-0.45)

* EPIC-InterAct, European Prospective Investigation into Cancer and Nutrition-InterAct study (n=27,296). Principal component analysis (PCA) was performed, evaluating 27 individual fatty acids among adults in a specific population sub-group (derivation sets) of the randomly-sampled sub-cohort population. Using the scoring coefficients in the derived pattern, the fatty acid pattern score was calculated among all adults in the validation set, not included in each derivation set. Thus, adults in the derivation set and validation set were mutually independent (with exception of the top row). In the validation set, prospective associations of the fatty acid pattern score with incident type 2 diabetes were examined by Cox proportional hazard regression.

† Pearson correlation coefficient between the two fatty acid pattern scores: one derived from all sub-cohort participants (top row), used in the main analysis, and the other one from each Derivation set.

‡ All estimates were for 10th to 90th percentile range determined by eight countries. The first model (the second right column) adjusted for recruitment centres (2 to 6 categories in each country), age, sex, education history (none, primary school, secondary school, technical school, university or upper), smoking status (never, former, current), alcohol consumption levels (continuous), dietary factors (dietary fibre, fruits, vegetables, processed meats, soft drinks), physical activity levels (inactive, moderately inactive, and moderately active, active), menopause status (pre-, peri- or post-menopause, or surgical post-menopause; women only), hormone replacement use (yes/no; women only), and prevalent diseases (yes, no for each myocardial infarction or angina, hypertension, and hyperlipidaemia). Sex was dropped, when used for stratification. The second model (the first right column) further adjusted for body-mass index, triglycerides and high-density lipoprotein cholesterol.

§ The HR (95% CI) derived in mutually independent strata were pooled by using random-effects meta-analysis.
