# Supplementary material for: A combination of plasma phospholipid fatty acids and its association with incidence of type 2 diabetes: The EPIC-InterAct case-cohort study
Source: PLoS Med. 2017 Oct 11;14(10):e1002409. doi: 10.1371/journal.pmed.1002409 (PMC5636062; doi:10.1371/journal.pmed.1002409)
Supplement: S5 Table — (PDF) [file pmed.1002409.s010.pdf]

S5 Table. Coefficients to calculate the fatty acid pattern score derived from the sub-cohort of EPIC-InterAct (n=15,919).*

Individual fatty acids	Name	Scoring coefficients †
Long-chain Saturated FA		
14:0	Myristic acid	-0.08608
16:0	Palmitic acid	-0.11792
18:0	Stearic acid	0.08241
Odd-chain saturated FA		
15:0	Pentadecanoic acid	0.05438
17:0	Heptadecanoic acid	0.12617
Very long-chain saturated FA		
20:0	Arachidic acid	0.11988
22:0	Behenic acid	0.14970
23:0	Tricosanoic acid	0.10356
24:0	Lignoceric acid	0.12923
Monounsaturated FA		
16:1	Palmitoleic acid	-0.17382
18:1 n-9	Oleic acid	-0.11268
Very long-chain monounsaturated FA		
20:1	Gondoic acid	-0.02372
24:1	Nervonic acid	0.10325
Omega-6 PUFA		
18:2 n-6	Linoleic acid	0.11022
18:3 n-6	γ-linolenic acid	-0.11696
20:3 n-6	Dihomo-γ-linolenic acid	-0.09440
20:4 n-6	Arachidonic acid	-0.04052
Omega-3 PUFA		
18:3 n-3	α-linolenic acid	-0.00800
20:5 n-3	Eicosapentaenoic acid	0.00527
22:5 n-3	Docosapentaenoic acid	-0.00351
22:6 n-3	Docosahexaenoic acid	0.05004
Trans unsaturated FA		
Trans 18:1	Elaidic acid	0.04266
Trans 18:2	Trans linoleic acid	0.03424
Others		
17:1	Heptadecenoic acid	-0.02832
20:2	Eicosadienoic acid	-0.02678
22:4	Adrenic acid	-0.09844
22:5 n-6	Docosapentaenoic acid	-0.09872

* EPIC-InterAct, European Prospective Investigation into Cancer and Nutrition-InterAct (EPIC-InterAct), using the fatty acid pattern score generated by applying principal component analysis to 27 individual fatty acids of circulating plasma phospholipids.

† A score can be generated by 1) multiplying each coefficient with each fatty acid scaled to be mean=0 and standard deviation=1; and 2) summing up the products.
