# Supplementary material for: A combination of plasma phospholipid fatty acids and its association with incidence of type 2 diabetes: The EPIC-InterAct case-cohort study
Source: PLoS Med. 2017 Oct 11;14(10):e1002409. doi: 10.1371/journal.pmed.1002409 (PMC5636062; doi:10.1371/journal.pmed.1002409)
Supplement: S1 Text — (PDF) [file pmed.1002409.s011.pdf]

S1 Text. Fatty acid assay and genetic information in EPIC-InterAct

Fatty acid assay in the EPIC-InterAct

The procedure, validation, and application to the EPIC-InterAct were documented in detail previously [1,2]. From plasma samples drawn at baseline and stored at -196°C (or -150 °C in Denmark), phospholipid fatty acids were extracted and isolated by centrifugation and solid-phase extraction. After hydrolysis of fatty acid moieties of phospholipids and esterification, more volatile fatty acid methyl esters (FAME) were formed, isolated, and injected to gas-chromatogram equipped with a column (J&W HP-88, 30m length, 0.25 mm internal diameter, 0.2 μm film thickness) and with flame ionisation detection (7890N GC Agilent Technologies, USA). Sample preparation, solid-phase phospholipid extraction, isolation of fatty acid moieties, esterification to FAME, and chromatographic assays were automated in sequential multipurpose sample system (Gerstel GmbH & Co. KG, Mülheim an der Ruhr, Germany). The overall system was confirmed to reduce laboratory errors, compared to measurements involving manual procedures [1]. Geometric information was insufficient to differentiate location of all double bonds. Thus, for example, *trans*-18:1 included mono-unsaturated fatty acid with trans conformation of a double bond located at either n-7, n-9, n-11, or others. We considered that concentrations of *trans*-18:1 represented total 18-carbon *trans* monounsaturated fatty acids.

We measured fatty acids with a total of 870 batches, where two quality control samples from pooled human plasma and equine plasma [1]. Samples from each country were measured in random and blinded manner. Non-randomness between countries could not influence our results, because we conducted all analyses stratified by country. We evaluated variability of the fatty acid pattern score across quality control samples. Assigning scoring coefficients of the principal component analysis to the quality control samples, the standard deviation was 0.06 and thus coefficient of variation was interpreted as 6% (as standard deviation of principal components are scaled to be 1.0).

Genetic information in the EPIC-InterAct

A maximum of 22,179 participants (12,885 from sub-cohorts; and 9,294 diabetes cases) had genotypes available from the Metabochip (N=13,013) at the Department of Pathology, University of Cambridge, Cambridge, UK, and Illumina 660W-Quad Chip (N=9,166) at the Sanger Institute (Hinxton, UK) imputed into 1000 Genomes and were included in the current study. We calculated weighted genetic risk scores for body-mass index (BMI) and insulin resistance based on single nucleotide polymorphisms (SNPs) identified to predict either trait in genome-wide association studies. For BMI, 97 SNPs were used as identified by Locke et al. [3]; for insulin resistance, 10 SNPs [4]. Reported additive effects of SNPs on BMI and on insulin resistance were used as weights multiplied with allelic information of participants and summed up as genetic risk scores for BMI and for insulin resistance, respectively. See "Cross-sectional Analysis" section for the information on the analysis relating the genetic risk scores to the fatty acid pattern score. As results from *post hoc* sensitivity analysis, the main effects of the fatty acid pattern score and of the genetic risk scores, evaluated previously [4], were materially unchanged when adjusted for each other mutually.

References

1. Wang L, Summerhill K, Rodriguez-Canas C, Mather I, Patel P, Eiden M, et al. Development and validation of a robust automated analysis of plasma phospholipid fatty acids for metabolic phenotyping of large epidemiological studies. Genome Med. 2013;5(4):39.

2. Forouhi NG, Koulman A, Sharp SJ, Imamura F, Kröger J, Schulze MB, et al. Differences in the prospective association between individual plasma phospholipid saturated fatty acids and incident type 2 diabetes: the EPIC-InterAct case-cohort study. Lancet Diab Endocrinol. 2014;2(10):810–8. doi:10.1016/S2213-8587(14)70146-9

3. Locke AE, Kahali B, Berndt SI, Justice AE, Pers TH, Day FR, et al. Genetic studies of body mass index yield new insights for obesity biology. Nature. 2015;518(7538):197–206. doi:10.1038/nature14177

4. Scott RA, Fall T, Pasko D, Barker A, Sharp SJ, Arriola L, et al. Common genetic variants highlight the role of insulin resistance and body fat distribution in type 2 diabetes, independent of

obesity. Diabetes. 2014;63(12):4378–87. doi:10.2337/db14-0319
