# Supplementary material for: A combination of plasma phospholipid fatty acids and its association with incidence of type 2 diabetes: The EPIC-InterAct case-cohort study
Source: PLoS Med. 2017 Oct 11;14(10):e1002409. doi: 10.1371/journal.pmed.1002409 (PMC5636062; doi:10.1371/journal.pmed.1002409)
Supplement: S2 Text — (PDF) [file pmed.1002409.s012.pdf]

**S2 Text. External validation of associations of a fatty acid pattern score**

We derived the scoring matrix to derive the fatty acid pattern score in the EPIC-InterAct and applied it to adults in the United States National Health and Nutrition Examination Survey, 2003-2004 (NHANES), adopting a method previously performed in analysis relating fatty acid patterns to cardiovascular outcomes [1]. In NHANES, fatty acid concentrations were measured among adult subsample (n=1,829). We excluded pregnant women (n=87) and adults with prevalent diabetes (n=176) and evaluated 1,566 adults in this study. Missing information was observed in fatty acid variables and other covariates (up to 6% of adults). The missing values were imputed by multivariable regression, after improving normality of distributions [2].

In NHANES, fatty acids were measured by mass-spectrometry using lipid fractions isolated from total plasma [3] which mean sum of free fatty acids and fatty acid esters in phospholipids, triglycerides, cholesteryl esters, and free fatty acids. Possible difference in fatty acid profiles between different lipid fractions and methods is of biological interest but beyond the scope of this study. However, differences were also considered unlikely to alter inter-relations between different fatty acids, which were our interest in the present study. Briefly, a fatty acid profile of total plasma may reflect hepatic synthesis of fatty acids more than a profile of phospholipids, because contributions by fatty acids of triglycerides and cholesteryl esters secreted from the liver [4]. In addition, phospholipid fatty acids had longer half-lives of fatty acids and reflect longer-term exposure to fatty acids than total plasma which include non-esterified fatty acids, considering a kinetic study of dietary fatty acids [4].

A fatty acid pattern score was calculated by multiplying each of scoring coefficients with each of a standardized variable and summing up the products. Thus, using results from principal components analysis, we extracted the scoring coefficients for 27 fatty acid variables in EPIC-InterAct. We applied the coefficients to fatty acid data in NHANES, after standardizing the variables within NHANES.

Individual fatty acids assessed varied between EPIC-InterAct (n=37 in total, 27 used for a fatty acid pattern) and NHANES (n=24). NHANES evaluated cis-18:1n-7 which was not assessed in EPIC-InterAct. We assumed 18:1n-9 in EPIC-InterAct was a sum of 18:1n-9 and 18:1n-7, and we summed concentrations of 18:1n-9 and 18:1n-7 in NHANES so that the variable represented the same 18-carbon monounsaturated fatty acid in the two datasets. In NHANES, 14:1n-5 was assessed (mean=0.06% of total), but not used as in EPIC-InterAct, because of the trace amount. Consequently, 22 fatty acids in NHANES were analysed in this study, not including odd-chain fatty acids (15:0, 17:0, and 23:0) and *trans* unsaturated fatty acids (trans-18:1, trans18:2) that were assessed in EPIC-InterAct. We thus used scoring coefficients for the 22 fatty acids, not 27, to calculate the fatty acid pattern score in NHANES. To validate the use of the smaller number of fatty acids, we re-derived a fatty acid pattern score in EPIC-InterAct and calculated the score in both EPIC-InterAct and NHANES, using 22 fatty acids rather than 27 fatty acids. In EPIC-InterAct, the correlation coefficient between the fatty acid pattern scores based on 22 fatty acids and that based on 27 fatty acids was 0.98 in all countries. Therefore, difference between using 27 fatty acids and 22 fatty acids is unlikely to have caused bias in our study.

**References**

1. Imamura F, Lemaitre RN, King IB, Song X, Lichtenstein AH, Matthan NR, et al. Novel circulating fatty acid patterns and risk of cardiovascular disease: the Cardiovascular Health Study. Am J Clin Nutr. 2012;96(6):1252–61. doi:10.3945/ajcn.112.039990

2. Jonathan ACS, Ian RW, John BC, Michael S, Patrick R, Michael GK, et al. Multiple imputation for missing data in epidemiological and clinical research: potential and pitfalls. BMJ. 2009;338(jun29 1):b2393–b2393. doi:10.1136/bmj.b2393

3. Lagerstedt SA, Hinrichs DR, Batt SM, Magera MJ, Rinaldo P, McConnell JP. Quantitative determination of plasma c8-c26 total fatty acids for the biochemical diagnosis of nutritional and metabolic disorders. Mol Genet Metab. 2001;73(1):38–45. doi:10.1006/mgme.2001.3170

4. Katan MB, Deslypere JP, van Birgelen AP, Penders M, Zegwaard M. Kinetics of the incorporation of dietary fatty acids into serum cholesteryl esters, erythrocyte membranes, and adipose

tissue: an 18-month controlled study. J Lipid Res. 1997;38(10):2012–22.
