# Supplementary material for: A combination of plasma phospholipid fatty acids and its association with incidence of type 2 diabetes: The EPIC-InterAct case-cohort study
Source: PLoS Med. 2017 Oct 11;14(10):e1002409. doi: 10.1371/journal.pmed.1002409 (PMC5636062; doi:10.1371/journal.pmed.1002409)
Supplement: S3 Text — (PDF) [file pmed.1002409.s013.pdf]

S3 Text. Cross-sectional analyses in EPIC InterAct and United States NHANES

Metabolic factors

Regression analyses were performed to examine relationships of the fatty acid pattern score with metabolic risk factors and with dietary factors. The results are presented in Table 3 and Figure 3. In EPIC-InterAct, estimates from each country were pooled by meta-analysis adjusting for each of potential confounders as shown in the footnote of Table 2. In the National Health and Nutrition Examination Survey (NHANES), weighting individuals by sampling probability, the model included selected potential confounders: age, gender, race (White, African American, Asian, Mexican Hispanic, other Hispanic), education (<high school, high school, >high school), annual family income (<$15,000, $15,000-$34,999, $35,000 or more), smoking status (never, former, current), alcohol (spline terms), prevalent diseases (positive for either coronary heart disease, hyperlipidaemia, or hypertension), family history of diabetes, and family history of myocardial infarction. Body-mass index (BMI) was additionally adjusted for in the analyses for metabolic risk factors, with exception of analysis of BMI. Risk for non-alcoholic fatty liver disease was evaluated by modified Poisson regression [1]. Analyses using different cutpoints for non-alcoholic fatty liver disease showed similar results.

Genetic factors

In the analysis to assess the association between genetic risk factors and the fatty acid pattern score, genetic variables were considered as causal determinants of the fatty acid pattern score. Therefore, genetic risk scores were treated as independent variables and the fatty acid pattern score was treated as a dependent variable. For interpretability, these variables were scaled so that a regression coefficient can be interpreted as % difference in the fatty acid pattern score per the interdecile range (10th and 90th percentile range). Regression results were obtained by country and pooled by random-effects meta-analysis. The measure of heterogeneity, I^2, was 0%, indicating no heterogeneity in the impact of the selected genetic effects on the fatty acid pattern. A few considerations were taken in regards to confounding. First, the analyses were performed with adjustment for potential confounders

as selected above and also for genotyping sources (metabochip or not). Second, as genetic variability was determined at the time of conception, any modifiable factors at cohort baseline could not be confounders. Thus, analyses were repeated with adjustment only for centres, age, sex, and genotyping sources (not shown because results indicated no meaningful impact). Third, in this case-cohort design [2], cases were oversampled, but there was little concern of reverse causality in this genetic analysis. Thus, analyses were performed, stratified by the cohort status (sub-cohort or not) and pooled by random-effects meta-analysis.

Dietary factors

We performed dietary analysis in each of EPIC-InterAct and NHANES to identify dietary exposure associated with the main fatty acid pattern identified in EPIC-InterAct. In EPIC-InterAct, we examined adults included in the sub-cohort. We evaluated adults with information both on fatty acid profiles and dietary consumption, retaining 15,566 adults in EPIC-InterAct (n excluded=353 from the sub-cohort) and 1,500 adults in NHANES (n excluded=66). In EPIC-InterAct, dietary variables were derived from responses to food-frequency questionnaires and dietary records in a standardized manner [3–5]. In NHANES, dietary information from two 24-hour dietary recalls was used [6,7]. In each study population, dietary factors were modelled as independent variables in linear regression analysis adjusting for potential confounders. In EPIC-InterAct, country-specific estimates were first obtained and pooled by random-effects meta-analysis.

In nutrient-based analysis, saturated fatty acids, monounsaturated fatty acids, polyunsaturated fatty acids, carbohydrates, and protein were evaluated. We considered that dietary fatty acids influence circulating fatty acids directly and carbohydrates and proteins from a diet are converted to fatty acids through enzymatic pathways including citric acid cycle and de novo lipogenesis [8]. Fibre intake was examined because of its association with type 2 diabetes and its effects on metabolism of lipids [9,10]. For specific dietary fat, carbohydrates, and protein, we performed isocaloric substitution analysis [11]. We fitted a multivariable-adjusted linear regression model to estimate hypothetical effects of replacing 5% of total calories from carbohydrates intakes with 5% of total calories from each type of

dietary fats and protein [11]. The model simultaneously included fibre intake and adjusted for socio-demographic variables, lifestyle factors, prevalent clinical characteristics, total energy intake, and consumption of alcoholic beverages which was examined in food-based analysis.

In food-based analysis, we performed analysis first in EPIC-InterAct and then selected a small subset of food variables that showed trends toward positive or negative associations with the fatty acid pattern score. Then, in NHANES, we examined the associations of the small subset of foods or beverages with the fatty acid pattern score. Variables evaluated in the EPIC-InterAct were selected on the basis of associations with fatty acids or type 2 diabetes or fatty acid profiles, including potatoes, rice or pasta, cereals, fruits, vegetables, nuts, legumes, vegetable oils, margarine, cheese, yoghurt, meats, processed meats, fish, soft drinks, coffee, tea, and alcoholic beverages [10,12–14]. We first evaluated each of these dietary factors without co-adjustment for each other (S4 Fig). To focus a fewer number of these dietary factors, we performed a variable selection procedure (backward selection) in EPIC-InterAct, using results from meta-analysis combining country-specific estimates for the food variables. In the backward selection, $p<0.2$ was used to keep variables showing significant associations or non-significant trends of associations; the p-value cutpoint was considered to be informative for descriptive purpose and important for adjustment for dietary confounding [15]. In NHANES, 4,573 food variables were compiled with 8-digit food code numbers assigned. Using the food code numbers and associated information [6], we created food variables that corresponded to a small subset of food variables identified in EPIC-InterAct. Including the food variables simultaneously in a multivariable-adjusted linear regression model, we examined associations of consumption of the foods with the fatty acid pattern score in NHANES.

For interpretability, the fatty acid pattern score was scaled to one standard deviation (SD) so that regression coefficient meant a difference of the fatty acid pattern score by percentages of one SD according to 1 unit difference in dietary variables. Each of dietary variables was also scaled so that 1 unit became practically informative (e.g. 1 serving per day).

References

1. Spiegelman D, Hertzmark E. Easy SAS calculations for risk or prevalence ratios and differences. American journal of epidemiology. 2005;162(3):199–200. doi:10.1093/aje/kwi188
2. Forouhi NG, Koulman A, Sharp SJ, Imamura F, Kröger J, Schulze MB, et al. Differences in the prospective association between individual plasma phospholipid saturated fatty acids and incident type 2 diabetes: the EPIC-InterAct case-cohort study. Lancet Diab Endocrinol. 2014;2(10):810–8. doi:10.1016/S2213-8587(14)70146-9
3. Riboli E, Hunt KJ, Slimani N, Ferrari P, Norat T, Fahey M, et al. European Prospective Investigation into Cancer and Nutrition (EPIC): study populations and data collection. Public Health Nutr. 2002;5(6b):1113–24. doi:doi:10.1079/PHN2002394
4. Margetts BM, Pietinen P. European Prospective Investigation into Cancer and Nutrition: validity studies on dietary assessment methods. Int J Epidemiol. 1997;26 Suppl 1(1):S1-5.
5. Slimani N, Deharveng G, Unwin I, Southgate DAT, Vignat J, Skeie G, et al. The EPIC nutrient database project (ENDB): a first attempt to standardize nutrient databases across the 10 European countries participating in the EPIC study. Eur J Clin Nutr. 2007;61(9):1037–56. doi:10.1038/sj.ejcn.1602679
6. USDA Food and Nutrient Database for Dietary Studies, 2.0 [Internet]. Beltsville, MD: Agricultural Research Service, Food Surveys Research Group; 2006.
7. Centers for Disease Control and Prevention (CDC), National Center for Health Statistics (NCHS), National Health and Nutrition Examination Survey Data, 2003-2004. [Internet].
8. Hodson L, Skeaff CM, Fielding BA. Fatty acid composition of adipose tissue and blood in humans and its use as a biomarker of dietary intake. Prog Lipid Res. 2008;47(5):348–80. doi:10.1016/j.plipres.2008.03.003
9. Topping DL, Clifton PM. Short-chain fatty acids and human colonic function: roles of resistant starch and nonstarch polysaccharides. Physiol Rev. 2001;81(3):1031–64.
10. Ley SH, Hamdy O, Mohan V, Hu FB, S.H. L, O. H, et al. Prevention and management of type 2 diabetes: dietary components and nutritional strategies. Lancet. 2014;383(9933):1999–2007. doi:10.1016/S0140-6736(14)60613-9

11. Willett WC, Howe GR, Kushi LH. Adjustment for total energy intake in epidemiologic studies. Am J Clin Nutr. 1997;65(4 Suppl):1220S–1228S; discussion 1229S–1231S.

12. The InterAct Consortium. Tea Consumption and Incidence of Type 2 Diabetes in Europe: The EPIC-InterAct Case-Cohort Study. PLoS One. 2012;7(5):e36910. doi:10.1371/journal.pone.0036910

13. Sluijs I, Forouhi NG, Beulens JWJ, van der Schouw YT, Agnoli C, Arriola L, et al. The amount and type of dairy product intake and incident type 2 diabetes: results from the EPIC-InterAct Study. Am J Clin Nutr. 2012;96(2):382–90.

14. Cooper AJ, Forouhi NG, Ye Z, Buijsse B, Arriola L, Balkau B, et al. Fruit and vegetable intake and type 2 diabetes: EPIC-InterAct prospective study and meta-analysis. Eur J Clin Nutr. 2012;66(10):1082–92. doi:10.1038/ejcn.2012.85

15. Maldonado G, Greenland S. Simulation Study of Confounder-Selection Strategies. Am J Epidemiol. 1993;138(11):923–36.
